# Supplementary material for: What happened to health labour markets during COVID-19? Insights from the analysis of cross-sectional survey data on the perceptions of medical doctors in Brazil
Source: BMJ Open. 2023 Aug 9;13(8):e075458. doi: 10.1136/bmjopen-2023-075458 (PMC10414124; doi:10.1136/bmjopen-2023-075458)
Supplement: Supplementary data [file bmjopen-2023-075458supp002.pdf]

Table S1: Physicians sample, but location and socio-economic characteristics

Physicians' sociodemographic characteristics	Proportion of total (n = 1,183)	São Paulo (n = 632) 53.4%	Maranhão (n = 551) 46.6%
Gender			
Male	56.2	54.1	58.6
Female	43.8	45.9	41.4
Age			
24 to 34	34.1	34.3	33.9
35 to 44	24.5	20.7	28.9
45 to 59	20.4	22.3	18.1
≥60	21.0	22.6	19.1
Geographical location of deployment			
Rural areas (Interior)	50.5	54.9	45.6
Urban areas around capital cities	49.5	45.1	54.5
Health sector of deployment			
Exclusively public	19.6	14.2	25.8
Exclusively private	22.1	31.5	11.3
Dual practice	58.3	54.3	63.0
Working directly with COVID-19 patients			
Yes	63.4	59.2	68.2
No	36.6	40.8	31.8

Table S2: Physicians' perceptions on employment opportunities in public and private facilities, by State

**Employment opportunities**		**Total (n=1,181)**		**São Paulo (n=632) 53.4% (50.6-56.3)**		**Maranhão (n=551) 46.6% (43.7-49.4)**	
		%	CI95%	%	CI95%	%	CI95%
**Public sector**							
SUS	Increased	72.2	(66.1-77.7)	67.4	(57.0-76.4)	75.2	(67.4-81.6)
	Reduced	11.9	(8.3-16.8)	18.6	(11.8-28.1)	7.8	(4.4-13.4)
	No changes	15.9	(11.7-21.2)	14.0	(8.2-22.8)	17.0	(11.7-24.1)
Private doctor's practice	Increased	18.8	(13.9-25.0)	13.0	(7.0-23.0)	22.2	(15.6-30.6)
	Reduced	55.4	(48.2-62.3)	65.2	(53.4-75.4)	49.6	(40.7-58.5)
	No changes	25.8	(20.1-32.5)	21.7	(13.6-32.8)	28.2	(20.8-37.0)
Large private hospitals	Increased	70.2	(63.5-76.1)	68.9	(57.7-78.3)	71.0	(62.4-78.2)
	Reduced	12.6	(8.7-18.0)	14.9	(8.5-24.7)	11.3	(6.9-18.1)

	No changes	17.2	(12.6-23.0)	16.2	(9.5-26.2)	17.7	(12.0-25.4)
Walk-in private clinics	Increased	40.9	(34.0-48.2)	45.9	(34.0-58.3)	38.3	(30.1-47.3)
	Reduced	21.0	(15.7-27.5)	21.3	(12.9-33.1)	20.8	(14.5-28.9)
	No changes	38.1	(31.4-45.4)	32.8	(22.3-45.3)	40.8	(32.5-49.8)
**Private sector**							
SUS	Increased	53.1	(46.4-59.6)	47.4	(39.7-55.3)	67.8	(55.1-78.3)
	Reduced	17.8	(13.3-23.5)	19.5	(14.0-26.4)	13.6	(7.0-24.5)
	No changes	29.1	(23.4-35.5)	33.1	(26.2-40.9)	18.6	(10.7-30.4)
Private doctor's practice	Increased	12.2	(8.7-16.7)	9.2	(5.9-14.0)	22.0	(13.4-34.1)
	Reduced	62.7	(56.7-68.4)	65.8	(58.9-72.1)	52.5	(40.0-64.7)
	No changes	25.1	(20.2-30.8)	25.0	(19.5-31.5)	25.4	(16.1-37.8)
Large private hospitals	Increased	49.6	(43.3-55.9)	45.2	(38.0-52.6)	62.7	(50.0-73.9)
	Reduced	24.2	(19.1-30.0)	26.0	(20.1-32.9)	18.6	(10.7-30.4)
	No changes	26.3	(21.1-32.2)	28.8	(22.6-35.9)	18.6	(10.7-30.4)
Walk-in private clinics	Increased	37.1	(30.3-44.5)	36.4	(28.6-45.0)	39.1	(26.4-53.5)
	Reduced	28.6	(22.4-35.7)	29.5	(22.3-37.8)	26.1	(15.6-40.3)
	No changes	34.3	(27.7-41.6)	34.1	(26.5-42.6)	34.8	(22.7-49.2)
**Dual practice**							
SUS	Increased	56.4	(52.6-60.1)	49.8	(44.4-55.3)	62.5	(57.3-67.5)
	Reduced	18.0	(15.3-21.1)	21.8	(17.6-26.6)	14.5	(11.1-18.6)
	No changes	25.6	(22.4-29.1)	28.3	(23.7-33.5)	23.0	(18.8-27.8)
Private doctor's practice	Increased	18.1	(15.4-21.2)	16.6	(12.9-21.0)	19.6	(15.7-24.2)
	Reduced	62.5	(58.8-66.1)	64.7	(59.4-69.7)	60.4	(55.1-24.5)
	No changes	19.3	(16.5-22.5)	18.7	(14.9-23.3)	19.9	(16.0-24.5)
Large private hospitals	Increased	46.3	(42.4-50.1)	43.4	(38.0-48.9)	49.1	(43.7-54.5)
	Reduced	32.2	(28.7-36.0)	36.1	(31.0-41.5)	28.5	(23.9-33.7)
	No changes	21.5	(18.5-24.8)	20.6	(16.5-25.4)	22.4	(18.2-27.2)
Walk-in private clinics	Increased	34.6	(30.4-39.1)	40.1	(33.6-47.0)	30.4	(25.1-36.2)
	Reduced	31.8	(27.7-36.2)	27.7	(22.0-34.3)	35.0	(29.5-41.0)
	No changes	33.5	(29.4-38.0)	32.2	(26.1-38.9)	34.6	(29.1-40.6)
**Total**							
SUS	Increased	59.0	(56.1-61.9)	51.9	(47.7-56.0)	66.4	(62.3-70.3)
	Reduced	16.7	(14.6-19.0)	20.7	(17.5-24.2)	12.6	(10.1-15.7)
	No changes	24.3	(21.8-26.9)	27.5	(23.9-31.3)	21.0	(17.7-24.6)
Private doctor's practice	Increased	15.8	(13.8-18.0)	13.7	(11.2-16.7)	20.5	(17.2-24.2)
	Reduced	57.4	(54.5-60.2)	65.1	(61.2-68.9)	57.0	(52.7-61.3)
	No changes	20.3	(18.1-22.7)	21.2	(18.0-24.6)	22.5	(19.1-26.3)

Hospitais Large private hospitals	Increased	51.4	(48.4-54.4)	47.3	(43.2-51.4)	56.0	(51.7-60.2)
	Reduced	26.9	(24.3-29.6)	30.2	(26.5-34.1)	23.2	(19.7-27.0)
	No changes	21.7	(19.4-24.3)	22.6	(19.3-26.2)	28.8	(17.5-24.6)
Walk-in private clinics	Increased	36.6	(33.3-39.9)	39.8	(35.1-44.7)	33.6	(29.2-38.2)
	Reduced	28.7	(25.7-31.9)	27.3	(23.1-31.9)	30.0	(25.9-34.6)
	No changes	34.7	(31.5-38.0)	32.9	(28.4-37.7)	36.4	(32.0-41.1)

Source: USP-UFMA-QMUL (2022)

Table S3: Perceptions on changes in remuneration for 12h A&E shift

Remuneration per shift		Total (n=1,181)		São Paulo (n=632) 53.4% (50.6-56.3)		Maranhão (n=551) 46.6% (43.7-49.4)	
		%	CI95%	%	CI95%	%	CI95%
Public	Increased	15.7	(11.5-21.2)	14.8	(8.7-24.1)	16.3	(11.0-23.4)
	Reduced	7.9	(5.0-12.2)	7.4	(3.4-15.2)	8.2	(4.6-14.0)
	No changes	39.3	(30.3-45.6)	44.7	(37.7-55.5)	43.2	(37.7-52.0)
	Unknown	37.1	(29.2-43.1)	33.1	(27.1-37.1)	42.3	(38.2-51.4)
Private	Increased	8.6	(5.5-13.2)	8.8	(5.3-14.2)	8.0	(3.2-18.8)
	Reduced	10.5	(7.0-15.4)	10.7	(6.8-16.5)	10.0	(4.4-21.4)
	No changes	40.9	(35.0-55.6)	40.5	(33.7-55.9)	42.0	(39.2-50.2)
	Unknown	40.0	(32.1-54.3)	37.3	(31.4-42.4)	42.3	(37.1-51.3)
Dual practice	Increased	12.4	(10.0-15.1)	12.8	(9.6-16.9)	11.9	(8.8-16.0)
	Reduced	14.9	(12.3-17.8)	16.2	(12.6-20.6)	13.5	(10.2-17.7)
	No changes	42.8	(39.2-46.1)	41.3	(35.8-45.7)	44.5	(39.5-49.0)
	Unknown	29.9	(21.3-33.4)	39.7	(33.6-43-4)	30.1	(25.5-36.1)
Total	Increased	12.3	(10.5-14.4)	11.9	(9.5-14.9)	12.7	(10.1-15.9)
	Reduced	12.6	(10.7-14.7)	13.4	(10.8-16.4)	11.7	(9.2-14.8)
	No changes	39.1	(32.4-47.6)	34.3	(28.9-48.1)	43.5	(36.6-48.1)
	Unknown	36.0	(28.7-42.6)	40.4	(32.4-45.8)	32.1	(25.2-37.8)

Source: USP-UFMA-QMUL (2022)

Figure S1: Geographical location of the surveyed physicians in São Paulo and Maranhão, Brazil

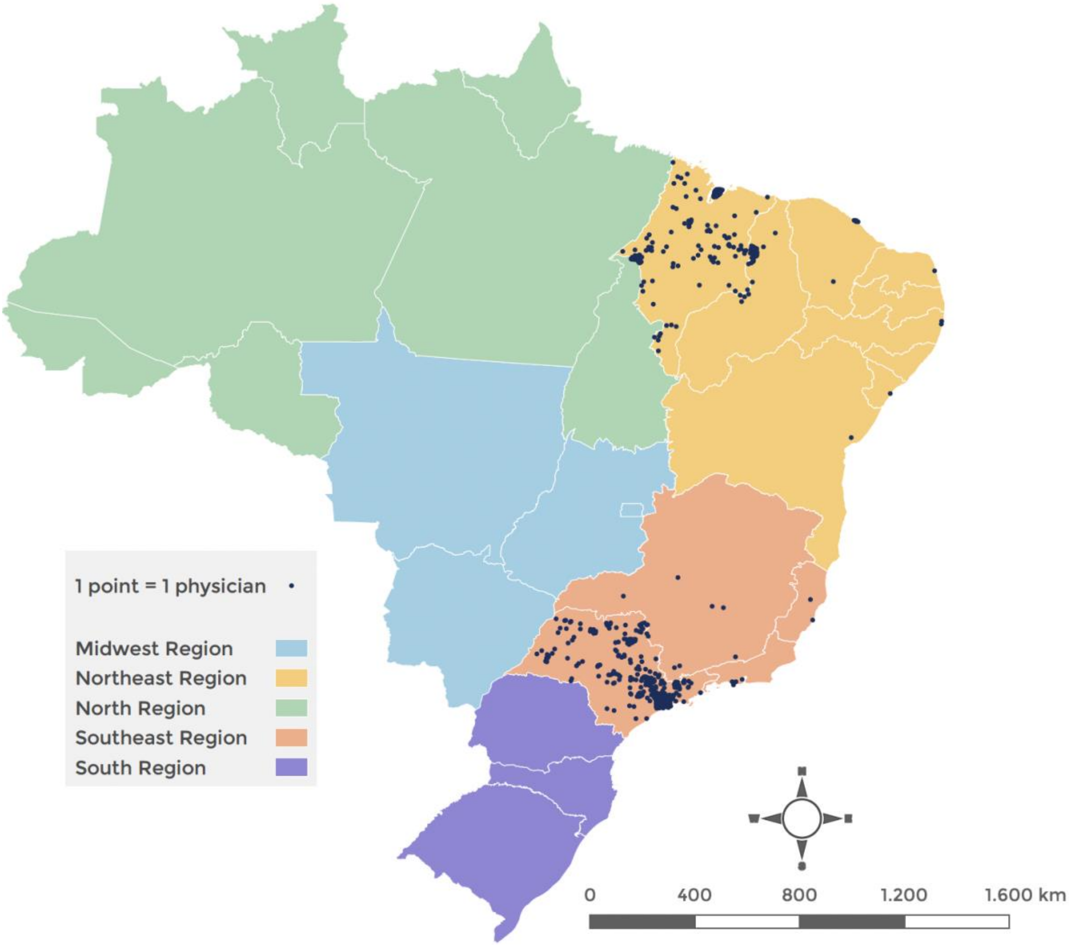

Source: Authors' own elaboration. The base layer map of Brazil is from the Brazilian Institute of Geography and Statistics.https://www.ibge.gov.br/geociencias/organizacao-do-territorio/malhas-territoriais/15774-malhas.html?=&t= downloads.
