# Supplementary material for: What happened to health labour markets during COVID-19? Insights from the analysis of cross-sectional survey data on the perceptions of medical doctors in Brazil
Source: BMJ Open. 2023 Aug 9;13(8):e075458. doi: 10.1136/bmjopen-2023-075458 (PMC10414124; doi:10.1136/bmjopen-2023-075458)
Supplement: Author's manuscript [file bmjopen-2023-075458.draft_revisions.pdf]

BMJ Open

BMJ Open is committed to open peer review. As part of this commitment we make the peer review history of every article we publish publicly available.

When an article is published we post the peer reviewers' comments and the authors' responses online. We also post the versions of the paper that were used during peer review. These are the versions that the peer review comments apply to.

The versions of the paper that follow are the versions that were submitted during the peer review process. They are not the versions of record or the final published versions. They should not be cited or distributed as the published version of this manuscript.

BMJ Open is an open access journal and the full, final, typeset and author-corrected version of record of the manuscript is available on our site with no access controls, subscription charges or pay-per-view fees (http://bmjopen.bmj.com).

If you have any questions on BMJ Open's open peer review process please email info.bmjopen@bmj.com

BMJ Open

What happened to health labour markets during COVID-19? Insights from a survey of medical doctors in Brazil

Journal:	*BMJ Open*
Manuscript ID	bmjopen-2023-075458
Article Type:	Original research
Date Submitted by the Author:	09-May-2023

Complete List of Authors:	Russo, Giuliano; Queen Mary University of London, Centre fo primary care and public health<br>de Oliveira, Bruno Luciano Carneiro Alves; Universidade Federal do Maranhao, Curso de Medicina<br>Cassenote, Alex; Universidade de São Paulo Hospital das Clínicas<br>Scheffer, Mário; Universidade de Sao Paulo Faculdade de Medicina, Preventative Medicine

Keywords:	COVID-19, Health economics < HEALTH SERVICES ADMINISTRATION & MANAGEMENT, Health policy < HEALTH SERVICES ADMINISTRATION & MANAGEMENT

SCHOLARONE™
Manuscripts

I, the Submitting Author has the right to grant and does grant on behalf of all authors of the Work (as defined in the below author licence), an exclusive licence and/or a non-exclusive licence for contributions from authors who are: i) UK Crown employees; ii) where BMJ has agreed a CC-BY licence shall apply, and/or iii) in accordance with the terms applicable for US Federal Government officers or employees acting as part of their official duties; on a worldwide, perpetual, irrevocable, royalty-free basis to BMJ Publishing Group Ltd ("BMJ") its licensees and where the relevant Journal is co-owned by BMJ to the co-owners of the Journal, to publish the Work in this journal and any other BMJ products and to exploit all rights, as set out in our licence.

The Submitting Author accepts and understands that any supply made under these terms is made by BMJ to the Submitting Author unless you are acting as an employee on behalf of your employer or a postgraduate student of an affiliated institution which is paying any applicable article publishing charge ("APC") for Open Access articles. Where the Submitting Author wishes to make the Work available on an Open Access basis (and intends to pay the relevant APC), the terms of reuse of such Open Access shall be governed by a Creative Commons licence – details of these licences and which Creative Commons licence will apply to this Work are set out in our licence referred to above.

Other than as permitted in any relevant BMJ Author's Self Archiving Policies, I confirm this Work has not been accepted for publication elsewhere, is not being considered for publication elsewhere and does not duplicate material already published. I confirm all authors consent to publication of this Work and authorise the granting of this licence.

For peer review only

Title Page

Title: What happened to health labour markets during COVID-19? Insights from a survey of medical doctors in Brazil

Running heading: Health labour market in Brazil during COVID-19

Authors' information

Giuliano Russo (corresponding author), Wolfson Institute of Population Health, Queen Mary University of London, United Kingdom. Email: g.russo@qmul.ac.uk. ORCID ID: 0000-0002-2716-369X

Bruno Luciano Carneiro Alves de Oliveira, Program in Public Health, Federal University of Maranhão, São Luís 65080-805, Brazil. ORCID ID: 0000-0001-8053-7972.

Alex Cassenote, Department of Preventive Medicine, University of São Paulo, Piracicaba 13416-000, Brazil. ORCID ID: 0000-0002-5098-1922.

Mário Scheffer. Department of Preventive Medicine, University of São Paulo, Piracicaba 13416-000, Brazil. ORCID ID: 0000-0001-8931-6471.

Abstract

Background. Limited evidence exists on impacts and adaptations of global health markets during COVID-19. We examined physicians' perceptions of changing employment opportunities in Brazil, to gain an insight into labour markets in low- and middle-income countries (LMICs) during the pandemic.

Methods. We conducted secondary analysis of a dataset from a representative cross-sectional survey of 1,183 physicians in São Paulo and Maranhão states in Brazil. We estimated prevalence and 95% Confidence Intervals (CI) for proxy variables of demand and supply of doctors, and prices of medical services for facilities of practice in the two States, stratified by public, private, and dual practice physicians.

Results. Most doctors reported increased job opportunities in the public sector (59.0%, 95% CI 56.1-61.9), particularly in Maranhão state (66.4%, 95% CI 62.3-70.3). For the private sector, increased opportunities were reported only in large private hospitals (51.4%, 95% CI 48.4-54.4), but not in smaller clinics. We recorded perceptions of slight increases in availability of doctors in Maranhão, particularly in the public sector (54.1%, 95 CI 45.7-62.3). Younger doctors recounted increased vacancies in the public sector (64%, 95 CI 58.1-68.1); older doctors only in walk-in clinics in Maranhão (47.5%, 95 CI 39.9-55.1). Those working directly with COVID-19 saw opportunities in public hospitals (65%, 95 CI 62.3-68.4), and in large private ones (55%, 95 CI 51.8-59.1)

Conclusions. Our findings suggest that health labour markets in LMICs may not necessarily shrink during epidemics, and that impacts will depend on the balance of public and private services in national health systems.

For peer review only

KEY MESSAGES

What is already known on this topic. Health labour markets are believed to shrink during epidemics, with fewer services and jobs available because of lockdowns and reduced demand.

What this study adds. The doctors we surveyed in Brazil noticed increased job opportunities in the public sector during COVID-19, particularly in Maranhão state. For the private sector, increased vacancies were reported in large private hospitals but not in smaller clinics.

How this study might affect research, practice or policy. The complementary roles of health markets and publicly or privately funded systems during a health emergency might need re-examining to improve pandemic preparedness in LMICs.

STATEMENTS AND DECLARATIONS

Competing Interests: The authors declare no competing interests.

ACKNOWLEDGMENTS

This study on whose findings this paper is based, received support from the Confap-MRC call for Health Systems Research Networks. Specifically, Bruno de Oliveira was funded by the Fundação de Pesquisa do Estado Maranhão (FAPEMA, Processo COOPI-00709/18) and Coordenação de Aperfeiçoamento de Pessoal de Nível Superior-Brasil (CAPES)—Finance Code 001, to the Graduate Program in Public Health. Giuliano Russo received an award from the NEWTON FUND, MEDICAL RESEARCH COUNCIL (UK), grant reference MR/R022747/1. Mário Scheffer and Alex Cassenote received an award from the Fundação de Amparo à Pesquisa do Estado de São Paulo (FAPESP-Brazil), 2017/50356-7.

For peer review only

For peer review only - http://bmjopen.bmj.com/site/about/guidelines.xhtml

Title: What happened to health labour markets during COVID-19? Insights from a survey of medical doctors in Brazil

ABSTRACT

Background. Limited evidence exists on impacts and adaptations of global health markets during COVID-19. We examined physicians' perceptions of changing employment opportunities in Brazil, to gain an insight into labour markets in low- and middle-income countries (LMICs) during the pandemic.

Methods. We conducted secondary analysis of a dataset from a representative cross-sectional survey of 1,183 physicians in São Paulo and Maranhão states in Brazil. We estimated prevalence and 95% Confidence Intervals (CI) for proxy variables of demand and supply of doctors, and prices of medical services for facilities of practice in the two States, stratified by public, private, and dual practice physicians.

Results. Most doctors reported increased job opportunities in the public sector (59.0%, 95% CI 56.1-61.9), particularly in Maranhão state (66.4%, 95% CI 62.3-70.3). For the private sector, increased opportunities were reported only in large private hospitals (51.4%, 95% CI 48.4-54.4), but not in smaller clinics. We recorded perceptions of slight increases in availability of doctors in Maranhão, particularly in the public sector (54.1%, 95 CI 45.7-62.3). Younger doctors recounted increased vacancies in the public sector (64%, 95 CI 58.1-68.1); older doctors only in walk-in clinics in Maranhão (47.5%, 95 CI 39.9-55.1). Those working directly with COVID-19 saw opportunities in public hospitals (65%, 95 CI 62.3-68.4), and in large private ones (55%, 95 CI 51.8-59.1)

Conclusions. Our findings suggest that health labour markets in (LMICs) may not necessarily shrink during epidemics, and that impacts will depend on the balance of public and private services in national health systems.

For peer review only

INTRODUCTION AND BACKGROUND

The COVID-19 pandemic and the ensuing economic crisis has had an unparalleled impact on population health [1], national economies [2], and working modalities [3]. A recent OECD report presents evidence on the ways labour markets have changed due to pandemic-related lockdown measures, as businesses were disrupted and workers furloughed or laid off [4].

The economic literature has so far suggested that health labour markets are 'recession proof', that is, one of the few sectors to hold up during economic slowdowns [5]. Scholars have argued this may be due to an inelastic demand for healthcare services, and to increasing health needs during recessions [6]. Evidence from the US in fact showed increasing employment opportunities in the healthcare industry during the 2008 financial crisis, particularly for nurses [7]. Studies from Canada and Australia concluded that labour markets for physicians were largely unaffected during the ensuing recession [8,9]. However, in those countries with publicly funded health systems, government-driven austerity measures did reduce health sector resources and shrink health labour markets, particularly in Europe [10–12].

There is a growing body of literature highlighting the role of labour markets in the provision of healthcare services in both high-income countries and LMICs [13,14], which tends to advocate for the use of Health Labour Market Analysis to design effective health policies in such contexts [15,16].

During COVID-19, research has shown that global labour health markets experienced a multi-layered crisis amplified by the combination of lockdown measures and reduced worker mobility [17]. In the first year of the pandemic, studies from the US argued that health sector unemployment rose because of pay cuts and redundancies, as patients were delaying seeking treatment, and hospitals were focussing on COVID-19 care, which "is not where the money is" [18]. Other US scholars [19] noticed that unemployment in the healthcare industry increased less than in other sectors during COVID-19; while less specialised jobs (such as non-healthcare hospital workers and therapists) were badly affected, employment opportunities for physicians barely shifted. As many patients were moved to government-funded schemes, new "price sensitivities" were found in the demand for healthcare service, as customers would no longer be insulated from costs [20].

The evidence from outside the US is less consolidated. Analysis of labour statistics and employment censuses from the UK market [21] show that healthcare employment declined suddenly in 2020 only to bounce back the year after, with dentists and nurses among the worst affected professions. Another British study looking at job ads during the pandemic [22] found more care work and nursing vacancies than in any other sector during lockdowns. Similar findings were reported for Serbia, where COVID-19 seems to have entrenched a continuous mismatch between supply and demand for physicians and nurses [23]. An online survey study from Iran [24] suggested that healthcare employment would have become less attractive because of COVID-19, as more health workers consider leaving. At the height of the first wave of the pandemic, there were reports [25] that Mexico, South Africa, and Zambia were recruiting doctors from abroad, as a surge in the demand for COVID-19 care was expected.

Brazil has been one of the world's most affected countries, with over 700,000 COVID-19 related deaths; its economy contracted by 3.9% in 2020, although it rebounded by 4.6% and 2.6% in the following years [26]. The country's unemployment rate is currently

For peer review only - http://bmjopen.bmj.com/site/about/guidelines.xhtml

at 9%, and the International Monetary Fund estimates that approximately 12 million jobs have been lost as a consequence of the pandemic, with a disproportionate impact among the lower income groups [27]. Past economic recessions historically had little repercussions for physician employment, as in Brazil demand has always outstripped supply [28]. But a recent study showed that public sector physicians' workload and earnings increased in Brazil during the first two years of the pandemic, while those of private doctors suffered [29].

Brazil's healthcare system comprises a publicly funded Unified Health System (*Sistema Único de Saúde*, SUS) and a multiplicity of privately financed sub-sectors, including large, comprehensive private hospitals and smaller, outpatient care surgeries [30]. In the last 20 years, low-cost walk-in clinics in urban areas (*Clínicas Populares*, or People's Clinics) have started to provide out-of-pocket private services, mostly outpatient in nature [31,32]. Within SUS, provision of free-at-the-point-of-care services is often outsourced to private entities (social health organisations) that manage public hospitals and contract their own staff, including doctors. Private funds account for more than half (54%) of Brazil's health spending, including out-of-pocket medicines and private insurance premiums [33]. Access to such private services is funded through employment-related health insurance plans, with 24.2% of Brazil's population owning such private plans [34].

This paper uses physicians' perceptions of changing employment opportunities in Brazil to gain an insight into health labour markets during COVID-19 in LMICs. The aim of this study is to contribute to the existing body of work on health labour markets in mixed health systems across the world; this would provide an evidence base for policies to mitigate the effects of future shocks on health workforces.

METHODS

Methodological approach

As part of a wider study on the health workforce in two Brazilian states [35], we conducted secondary analysis of a dataset from a representative cross-sectional survey on physicians' perceptions of the impact of COVID-19 on their health, earnings, and work routines [29,36,37]. Workers' perceptions have been used before in the economic literature to explore labour market dynamics in high-income contexts [38].

In our survey, doctors were asked whether they had observed in the past two years: (a) an increase or a decline of job opportunities and vacancies in their workplace; (b) any change in the availability of doctors; and (c) any change in the remuneration of a 12-hour shift in their institution's Accidents and Emergency (A&E) ward (see the Survey Questionnaire in Supplementary Information 1). We interpreted the reported changes in (a) as proxies for changes in demand for doctors; responses on (b) as proxies for supply; and responses on (c) as a proxy for changes in the level of prices for healthcare services.

As our doctors worked in either public or private facilities, or both, we considered the doctors' perceptions of changes in their own sector of employment as particularly accurate. As a way of validating responses, we triangulated the perceptions of the entire sample with those from doctors working specifically in such sectors. We outline the limitations of such an approach in the Discussion.

For peer review only - http://bmjopen.bmj.com/site/about/guidelines.xhtml

Study settings and data collection

The original survey was carried out in one rich state (São Paulo) and one less developed one (Maranhão) in Brazil, with a view to capture the differential effects of economic recessions in diverse health markets [34]. São Paulo state is home to more than 46.6M people, has one of the country's highest per capita incomes, and 38% of its population is covered by private health schemes. The public health expenditure is also among the highest, estimated at US$ 360,28 per capita in 2018 (US$ 650 PPP), and its medical workforce includes 163,430 physicians (3.5 per 1,000 inhabitants, the third highest in the country). By contrast, Maranhão state is home to approximately 7.2M people, its per capita income is one-third of São Paulo's, and only 1% of the population is covered by private health schemes. In 2022, there were 8,743 physicians in Maranhão, that is, 1.22 per 1,000 – the second lowest rate among Brazilian states [39].

Our survey was conducted between 16 February and 15 June 2021. The sample was drawn from the nominal listing of physicians registered with Brazil's Federal Council of Medicine in the two states. The study's overall sample was composed of 1,183 physicians, consisting of 632 from São Paulo and 551 from Maranhão. The sample was calculated based on the active physicians registered with the Federal Council of Medicine in the two states in 2021 (N=152,511 – 144,852 in São Paulo and 7,659 in Maranhão) and their key demographic characteristics. Proportional stratified sampling was used to replicate the physician distribution by gender, age, state, and residence. A larger proportion of Maranhão's universe of physicians was selected to allow for a sufficient N for the strata and doctor characteristics of interest. As the two states are very different—in São Paulo there are 30% of all the physicians in Brazil, and in Maranhão a little more than 1%—a proportional sample would have been too small to allow stratifications in Maranhão (see Table 1 in Supplementary Information 2).

The survey was carried out by a specialised research institute (Datafolha Research Institute), under the technical supervision of the academic researchers. Primary data was collected via a telephone survey conducted in Portuguese by Datafolha data collectors, which included a field coordinator, experienced interviewers, and administrative staff responsible for checking missing data. Sample size calculations, sample selection, questionnaire design, substitution control, database assembly, and data analysis were performed by the authors of this paper.

The original survey received approval from the Research Ethics Committees of the Federal University of Maranhão, Brazil (CEP UFMA 3.051.875) and from the Faculty of Medicine of the University of São Paulo, Brazil (CEP FMUSP 3.136.269).

Patient and public involvement. Medical doctors as well as members of the public were consulted and participated in the design of the original version of the survey questionnaire. The questionnaire was then piloted by Datafolha in a subset of ten doctors in the two states, and a final version was elaborated following the feedback received.

Variables and data analysis

The interviews consisted of a 30-minute telephone questionnaire, containing 30 questions ranging from multiple, closed questions to interdependently concatenated and semi-open questions. The specific variables for this secondary analysis were constructed from the questions below from the Survey Questionnaire's Section III – Changes in the Labour Market (see Survey Questionnaire in Annex 1).

For peer review only - http://bmjopen.bmj.com/site/about/guidelines.xhtml

Table 1: Variables, survey questions, and categories used in the analysis

Variable	Survey question	Categories
Demand for physicians in the public sector (SUS)	Q.24 In your opinion, comparing to before the pandemic, at SUS____	1. There are fewer work opportunities<br>2. There have been no significant changes in work opportunities OR<br>3. There are more work opportunities?<br>9999. Does not know
Demand for physicians in private doctor surgeries	Q.26 In your opinion, comparing to before the start of the pandemic, in private doctor surgeries ____	1. There are fewer work opportunities<br>2. There have been no significant changes in work opportunities OR<br>3. There are more work opportunities?<br>9999. Does not know
Demand for physicians in large private hospitals	Q.28 In your opinion, comparing to before the start of the pandemic, in large private hospitals ____	1. There are fewer work opportunities<br>2. There have been no significant changes in work opportunities OR<br>3. There are more work opportunities?<br>9999. Does not know
Demand for physicians in private walk-in clinics	Q.30 In your opinion, comparing to before the start of the pandemic, in private walk-in clinics ____	1. There are fewer work opportunities<br>2. There have been no significant changes in work opportunities OR<br>3. There are more work opportunities?<br>9999. Does not know
Supply of physicians	Q.32 In your opinion, comparing to before the pandemic, the availability of physicians for recruiting _______	1. Has decreased<br>2. There have been no significant changes in the number of medical professionals available OR<br>3. Has increased?<br>9999. Does not know
Prices of healthcare services	Q.34 In your opinion, comparing to before the pandemic, the amount paid to physicians for a 12h A&E shift _______	1. Has decreased.<br>2. There have been no significant changes in the amount paid to physicians in your specialty OR<br>3. Has increased?<br>9999. Does not know

Source: USP-UFMA-QMUL (2022).

For our analysis in this study, the prevalence and 95% CIs of variables related to physicians' perceptions on changes in job opportunities and availability of doctors were estimated for the two states in their facilities of practice (public hospitals-SUS, private doctor's surgeries, large private hospitals, and walk-in clinics), and stratified for public-only physicians, private-only ones, and dual practitioners (Table 1).

Statistically significant differences at the 5% confidence level were considered in the absence of overlapping 95% CIs. Prevalence and 95 CIs for such perceptions were also analysed and plotted by physicians' age groups (24–34; 35–44; 45–59; 60+) and by their specific involvement with COVID-19 services. The database developed in Excel by the Datafolha data collectors was exported to R-Studio version 4.1.3 for statistical treatment [40].

For peer review only - http://bmjopen.bmj.com/site/about/guidelines.xhtml

RESULTS

Most doctors in our sample said job opportunities and vacancies in the public sector (SUS) increased during COVID-19 (59.0%, 95% CI 56.1-61.9). This was particularly evident among Maranhão doctors (66.4%, 95% CI 62.3-70.3). Opportunities and vacancies were also said to have increased in large private hospitals with in-patient care capacity (51.4%, 95% CI 48.4-54.4), although not as much as in SUS. Again, such positive perceptions were found to be more pronounced among Maranhão doctors (see Table 2).

Table 2: Physicians' perceptions on employment opportunities in public and private facilities, by sector of employment and state

Employment opportunities		Total (n=1,181)		São Paulo (n=632) 53.4% (50.6-56.3)		Maranhão (n=551) 46.6% (43.7-49.4)	
		%	IC95%	%	IC95%	%	IC95%
Public system (SUS)	Increased	59.0	(56.1-61.9)	51.9	(47.7-56.0)	66.4	(62.3-70.3)
	Reduced	16.7	(14.6-19.0)	20.7	(17.5-24.2)	12.6	(10.1-15.7)
	No changes	24.3	(21.8-26.9)	27.5	(23.9-31.3)	21.0	(17.7-24.6)
Private doctor's practices	Increased	15.8	(13.8-18.0)	13.7	(11.2-16.7)	20.5	(17.2-24.2)
	Reduced	57.4	(54.5-60.2)	65.1	(61.2-68.9)	57.0	(52.7-61.3)
	No changes	20.3	(18.1-22.7)	21.2	(18.0-24.6)	22.5	(19.1-26.3)
Large private hospitals	Increased	51.4	(48.4-54.4)	47.3	(43.2-51.4)	56.0	(51.7-60.2)
	Reduced	26.9	(24.3-29.6)	30.2	(26.5-34.1)	23.2	(19.7-27.0)
	No changes	21.7	(19.4-24.3)	22.6	(19.3-26.2)	28.8	(17.5-24.6)
Walk-in clinics	Increased	36.6	(33.3-39.9)	39.8	(35.1-44.7)	33.6	(29.2-38.2)
	Reduced	28.7	(25.7-31.9)	27.3	(23.1-31.9)	30.0	(25.9-34.6)
	No changes	34.7	(31.5-38.0)	32.9	(28.4-37.7)	36.4	(32.0-41.1)

Source: USP-UFMA-QMUL (2022).

For other private sector facilities, perceptions of changing employment opportunities were either negative or neutral, particularly for smaller private doctor's practices, where 57.4% of doctors declared opportunities to have actually decreased (95% CI 54.5-60.2). Such negative perceptions for smaller private clinics were especially acute in Maranhão (65.1%, 95% CI 61.2-68.9) (Table 2).

These perceptions were confirmed when separating the views of public and private sector doctors, as public doctors declared noticing increased opportunities in their own sector of employment by a larger margin (72.2%, 95% CI 66.1-77.7) and private sector doctors confirmed the reduction of opportunities in private doctor's practices by 62.7% (95% CI 56.7-68.4). For those doctors working concomitantly in public and private facilities – the dual practitioners – job opportunities increased in the public sector and in large private hospitals (56.4% and 46.3%, respectively), but reduced in smaller doctor's practices (62.5%, 95 CI 58.8-66.1) and stayed unchanged in walk-in clinics (see Table 2 in Supplementary Information 2).

For peer review only - http://bmjopen.bmj.com/site/about/guidelines.xhtml

For peer review only

In regard to the availability of doctors to take up vacancies in specific sectors, there was a slight perception of increased availability among public health physicians (44.8%, 95 CI 38.4-51.4), although this was predominantly driven by the positive perceptions of Maranhão doctors (54.1%, 95 CI 45.7-62.3) – among São Paulo doctors this perception was, in fact, neutral (see Table 3 below). Perceptions of increased availability of doctors were also recorded among dual practitioners in Maranhão (45%, 95 CI 39.7-50.4). For private health physicians, perceptions of positive changes were only significant for Maranhão doctors (46.6%, 95 CI 34.3-59.2).

Table 3: Perceptions on availability of doctors in each sector, by type of current public, private, and dual employment

Current sector of employment	Perception of availability of doctors	Total (n=1,181)		São Paulo (n=632) 53.4% (50.6-56.3)		Maranhão (n=551) 46.6% (43.7-49.4)	
		%	IC95%	%	IC95%	%	IC95%
Public sector (SUS)	Increased	44.8	(38.4-51.4)	30.2	(21.5-40.6)	54.1	(45.7-62.3)
	Reduced	27.7	(22.6-33,4)	39.5	(29.9-50.1)	28.1	(21.2-36.3)
	No changes	19.2	(14.9-24.5)	30.2	(21.5-40.6)	17.8	(12.2-25.1)
Private	Increased	41.2	(35.1-47.5)	39.4	(32.6-46.7)	46.6	(34.3-59.2)
	Reduced	25.6	(20.5-31.5)	27.8	(21.8-34.7)	19	(10.9-30.9)
	No changes	33.2	(27.5-39.4)	32.8	(26.3-39.9)	34.5	(23.6-47.3)
Dual practice	Increased	42.2	(38.4-46.0)	39.2	(34.0-44.7)	45	(39.7-50.4)
	Reduced	32.5	(29.0-36.2)	31.6	(26.8-37.0)	33.2	(28.4-38.5)
	No changes	25.3	(22.1-28.8)	29.1	(24.4-34.3)	21.8	(17.6-26.5)
Total	Increased	42.5	(39.6-45.4)	38.0	(34.1-42.0)	47.5	(43.3-51.8)
	Reduced	31	(28.4-33.8)	31.6	(28.0-35.5)	30.3	(26.6-34.4)
	No changes	26.5	(24.0-29.2)	30.4	(26.8-34.3)	22.1	(18.8-25.9)

Source: USP-UFMA-QMUL (2022).

Such reported changes in availability of vacancies and doctors, however, were not reflected in the perception of changes in prices; the overwhelming majority of doctors across all sectors (75.1%, 95 CI 72.4-77.6) declared that remuneration for a 12-hour A&E shift stayed broadly unchanged during the pandemic (see Table 3 in Supplementary Information 2).

When disaggregating responses by age groups, younger doctors (aged 24–34) were the ones declaring increased job opportunities, particularly in the public sector (64%, 95 CI 58.1-68.1) (see Figure 1 below).

Figure 1: Proportion of doctors reporting increased job opportunities, by age group and type of facility

Source: USP-UFMA-QMUL (2022).

For peer review only

In private walk-in clinics, however, it was only the older doctors (>60 years) who reported significantly improved work opportunities (47.5%, 95 CI 39.9-55.1), with all the other age groups reporting either decreased or unchanged employment opportunities (Figure 1).

Doctors working directly with COVID-19 cases generally reported increased opportunities, particularly in public hospitals (65%, 95 CI 62.3-68.4) and in large private ones (55%, 95 CI 51.8-59.1) (see Figure 2). Walk-in clinics were the only exceptions, as in such facilities specialising in working with COVID-19 did not appear to significantly increase job opportunities (35.9%, 95 CI 32.1-40.0).

Figure 2: Proportion of doctors declaring increased opportunities, by specialisation in COVID-19 cases and type of facility

Source: USP-UFMA-QMUL (2022).

For peer review only

Discussion

In our survey of labour market perceptions during COVID-19 among physicians in Brazil, we found that most doctors recounted increased job opportunities in the public sector, particularly in Maranhão state. For the private sector, perceptions were mixed, as increased opportunities were reported in large private hospitals but not in smaller practices or walk-in clinics. In regard to the availability of doctors, our survey recorded perceptions of small increases in Maranhão, particularly in the public sector. Remuneration of A&E shifts stayed broadly unchanged. Younger doctors were the ones declaring more job opportunities in public facilities. Older ones reported opportunities in walk-in clinics, particularly in Maranhão. Those doctors working directly with COVID-19 patients saw increases in SUS and large private hospitals, but not elsewhere.

We acknowledge that different doctors across the world experienced COVID-19 in a different way – from the frontline intensive care and infective diseases specialists who found themselves in the eye of the storm, to primary care specialists who transitioned to remote working and telemedicine, to surgeons who simply saw their non-essential procedures cancelled. However, our survey of physicians' perceptions in Brazil during COVID-19 suggests that job opportunities actually increased in the public sector and in large private hospitals. This is contrast to what was observed for US hospitals during the pandemic [20].

Our interpretation is that, in countries like Brazil with publicly funded health systems, resources (and jobs) were proactively redirected toward COVID-19 cases. This would explain why SUS and large private hospitals with in-patient care capacity in Brazil appear to have experienced additional vacancies to meet the increased demand for COVID-19 care. Conversely, smaller private health facilities with mostly outpatient capacity may have temporally suspended some of their operations during the pandemic, in connection with the slow-down of demand for elective procedures. This would be consistent with what was observed in public health systems in European countries [22].

Demand and supply for doctors in São Paulo and Maranhão appear to have experienced opposite pandemic effects, with increasing opportunities in the latter state

For peer review only - http://bmjopen.bmj.com/site/about/guidelines.xhtml

but not in the former. This may be due to the comparatively greater weight of public health services in Maranhão. In São Paulo, the private healthcare sector is very developed [41], with an estimated 86% of doctors engaging with it either exclusively or as dual practitioners [42]. We conclude that the São Paulo health labour market experienced during COVID-19 effects similar to the market in the US, while Maranhão's displayed features more like the European, Canadian, or Australian markets. Such effects were probably exacerbated by the scarcity of doctors in Maranhão [43], who inevitably ended up taking more responsibilities (and risks) in the COVID-19 fight [36].

Younger doctors reported increasing job opportunities across the board, particularly in the public sector and in Maranhão. We believe this reflects the decisions taken in Brazil – like in other countries – to deploy younger (and therefore less at risk) cadres to staff COVID-19 services and shelter more senior ones [36]. This would also be consistent with our findings on the increased opportunities in COVID-19 wards for younger doctors. We interpret the reported increase of opportunities for older doctors in walk-in clinics as an indication that more lucrative parts of the private market would still be primarily accessible to more senior, established physicians, with fewer opportunities for younger doctors [15].

Reports of no changes in remuneration for A&E shifts appear to be at odds with standard economic theory that would predict an increase of prices in the presence of increased demand and stable supply of doctors [44]. On the one hand, we acknowledge that remuneration for 12-hour A&E shifts may not be a suitable bell weather for changes in equilibrium prices for medical services (see the Limitations section below). On the other hand, it may be possible that, in the short run, labour prices for medical services may prove inelastic [45], particularly during a pandemic emergency.

Our findings have broader relevance for other countries and future epidemics. We showed that health labour markets do not necessarily shrink during outbreaks, and the impacts will depend on the balance of public and private services within national health systems. Public health systems (and physicians) around the world were a key pillar of policy response to the pandemic, opening new services, performing additional functions, and driving the clinical fight [46]. This inevitably poses questions on what the role of markets and the private sector should be during a public health emergency [47], calling for a re-configuration of the complementarity of public and private functions, with a view to boosting pandemic preparedness in LMICs [48].

Limitations

Our findings need to be interpreted in light of a number of limitations. First, we used physicians' perceptions of changes in vacancies and prices to gain an insight into the demand and supply of doctors during the pandemic in Brazil. Although workers' perceptions have been used before in the literature to explore labour market dynamics [38], we acknowledge that an examination of employment data would be needed to validate our findings. We also asked our sampled physicians to report on changes that happened in the past two years, which could have been affected by recall bias [49].

Secondly, our proxies for demand, supply, and price levels may have left too much room for interpretation, as some of our doctors struggled to distinguish between 'availability of job opportunities' and 'availability of doctors to take up jobs'. Our question on changes in remuneration for A&E shifts was driven by the need to identify a price indicator for medical services that could be known to all the doctors surveyed

For peer review only - http://bmjopen.bmj.com/site/about/guidelines.xhtml

[50]. However, we realise that not all doctors in Brazil carry out A&E shifts or necessarily have knowledge of changes in this price.

Finally, we recognise that Maranhão and São Paulo states present very particular configurations of labour market characteristics, organisation of health services, policies, and health workforces [29,34,39]. Therefore, our findings may not be entirely generalisable to other LMICs.

Conclusion

Limited evidence exists on health labour markets' impacts and adaptations during COVID-19, with some literature suggesting a reduction of services. We conducted a secondary analysis of survey data on physicians' perceptions around changing employment opportunities in one rich and one less developed state in Brazil in 2021, with the objective of gaining insights into health labour markets during epidemics in LMICs.

Most of our sampled doctors noticed increased job opportunities in the public sector, particularly in Maranhão state. For the private sector, perceptions were mixed, as increased opportunities were reported in large private hospitals but not in smaller clinics. Younger doctors perceived an increase of vacancies in public and in large private hospitals, while older ones reported opportunities in walk-in clinics, particularly in Maranhão. Those doctors working directly with COVID-19 patients saw increases in public and large private hospitals, but not elsewhere.

Our findings suggest that health labour markets may not necessarily shrink during epidemics, and that the impacts will depend on the balance of public and private services in national health systems. The complementary roles of health markets and of publicly and privately funded systems during a health emergency should be re-examined, with the objective of improving pandemic preparedness, particularly for LMICs.

For peer review only

REFERENCES

1 Wang H, Paulson KR, Pease SA, *et al.* Estimating excess mortality due to the COVID-19 pandemic: a systematic analysis of COVID-19-related mortality, 2020–21. *The Lancet* 2022; **399**: 1513–36.

2 Shang Y, Li H, Zhang R. Effects of Pandemic Outbreak on Economies: Evidence From Business History Context. *Frontiers in Public Health* 2021; **9**. https://www.frontiersin.org/articles/10.3389/fpubh.2021.632043 (accessed March 24, 2023).

3 Lee S, Schmidt-Klau D, Verick S. The Labour Market Impacts of the COVID-19: A Global Perspective. *Ind J Labour Econ* 2020; **63**: 11–5.

4 OECD. Labour market developments: The unfolding COVID-19 crisis. Paris: OECD, 2021 DOI:10.1787/7e1e1ad3-en.

5 Russo G, Silva TJ, Gassasse Z, Filippon J, Rotulo A, Kondilis E. The impact of economic recessions on health workers: a systematic review and best-fit framework synthesis of the evidence from the last 50 years. *Health Policy Plan* 2021; **36**: 542–51.

6 Ellis RP, Martins B, Zhu W. Health care demand elasticities by type of service. *J Health Econ* 2017; **55**: 232–43.

7 Staiger DO, Auerbach DI, Buerhaus PI. Registered Nurse Labor Supply and the Recession — Are We in a Bubble? *New England Journal of Medicine* 2012; **366**: 1463–5.

8 Chen A, Sasso AL, Richards MR. Graduating into a downturn: Are physicians recession proof? *Health Econ* 2018; **27**: 223–35.

9 Lavergne MR, Hedden L, Law MR, McGrail K, Ahuja M, Barer M. The impact of the 2008/2009 financial crisis on specialist physician activity in Canada. *Health Econ* 2018; **27**: 1859–67.

10 Galbany-Estragués P, Nelson S. Migration of Spanish nurses 2009-2014. Underemployment and surplus production of Spanish nurses and mobility among Spanish registered nurses: A case study. *Int J Nurs Stud* 2016; **63**: 112–23.

11 Bambra C, Garthwaite K, Copeland A, Barr B. All in it together? Health inequalities, austerity, and the 'Great Recession'. Oxford University Press, 2015 https://www.oxfordscholarship.com/view/10.1093/acprof:oso/9780198703358.001.0001/acprof-9780198703358-chapter-12 (accessed April 29, 2020).

12 Russo G, Pires CA, Perelman J, Gonçalves L, Barros PP. Exploring public sector physicians' resilience, reactions and coping strategies in times of economic crisis; findings from a survey in Portugal's capital city area. *BMC Health Serv Res* 2017; **17**: 207.

13 McPake B, Maeda A, Araújo EC, Lemiere C, El Maghraby A, Cometto G. Why do health labour market forces matter? *Bull World Health Organ* 2013; **91**: 841–6.

For peer review only

For peer review only - http://bmjopen.bmj.com/site/about/guidelines.xhtml

14 Scheffler RM, Herbst CH, Lemiere C, Campbell J. Health Labor Market Analyses in Low- and Middle-Income Countries: An Evidence-Based Approach. The World Bank, 2016 DOI:10.1596/978-1-4648-0931-6.

15 McPake B, Scott A, Edoka I. Analyzing Markets for Health Workers: Insights from Labor and Health Economics. The World Bank, 2014 DOI:10.1596/978-1-4648-0224-9.

16 WHO. Health labour market analysis guidebook. Geneva, Switzerland: Health Workforce Department, The World Health Organization, 2021.

17 Causa O, Abendschein M, Luu N, Soldani E, Soriolo C. The post-COVID-19 rise in labour shortages. Paris: OECD, 2022 DOI:10.1787/e60c2d1c-en.

18 Bhandari N, Batra K, Upadhyay S, Cochran C. Impact of COVID-19 on Healthcare Labor Market in the United States: Lower Paid Workers Experienced Higher Vulnerability and Slower Recovery. *Int J Environ Res Public Health* 2021; **18**: 3894.

19 Matta S, Nicholas LH. Changes in Unemployment Among Health Care Workers Following the COVID-19 Pandemic. *JAMA* 2022; **328**: 1639–41.

20 Teasdale B, Schulman KA. Are U.S. Hospitals Still “Recession-proof”? *N Engl J Med* 2020; **383**: e82.

21 Cantor J, Whaley C, Simon K, Nguyen T. US Health Care Workforce Changes During the First and Second Years of the COVID-19 Pandemic. *JAMA Health Forum* 2022; **3**: e215217.

22 Arthur R. Studying the UK job market during the COVID-19 crisis with online job ads. *PLOS ONE* 2021; **16**: e0251431.

23 Šantrić Milićević M, Mandić-Rajčević S, Stevanovic A. Health workers labor market before and during the Covid-19 pandemic: Health sector capacity of Serbia. *European Journal of Public Health* 2022; **32**: ckac131.283.

24 AL-Abrrow H, Al-Maatoq M, Alharbi RK, *et al.* Understanding employees’ responses to the COVID-19 pandemic: The attractiveness of healthcare jobs. *Global Business and Organizational Excellence* 2021; **40**: 19–33.

25 Shuchman M. Low- and middle-income countries face up to COVID-19. *Nature Medicine* 2020; **26**: 986–8.

26 The World Bank. Brazil Poverty and Equity Assessment : Looking Ahead of Two Crises. Washington (DC): The World Bank, 2022 https://documents.worldbank.org/en/publication/documents-reports/documentdetail/099230007062256153/P1746910e33a8407d0b0850b8f0f5bcf18c (accessed March 23, 2023).

27 Flamini V, Toscani F. The Short-Term Impact of COVID-19 on Labor Markets, Poverty and Inequality in Brazil. *IMF Working Papers* 2021. DOI:https://doi.org/10.5089/9781513571645.001.

For peer review only

For peer review only - http://bmjopen.bmj.com/site/about/guidelines.xhtml

28 Nogueira RP. [The socioeconomic crisis and the demand for health professionals in Brazil, 1976-1984]. *Bol Oficina Sanit Panam* 1988; **104**: 572–82.

29 de Oliveira BLCA, Andrietta LS, Reis RS, *et al.* The Impact of the COVID-19 Pandemic on Physicians' Working Hours and Earnings in São Paulo and Maranhão States, Brazil. *International Journal of Environmental Research and Public Health* 2022; **19**: 10085.

30 OECD. OECD Reviews of Health Systems: Brazil 2021. OECD, 2021 DOI:10.1787/146d0dea-en.

31 Godoy CV, Silva JB. O Fenômeno de expansão das "clínicas médicas populares" no bairro Centro de Fortaleza/Ceará/Brasil. In: XIV Colóquio Ibérico de Geografia. Fotaleza, Ceará, 2014. http://www.lasics.uminho.pt/conferences/index.php/CEGOT/XIV_ColoquioIbericoGeografia/paper/view/1963 (accessed May 8, 2020).

32 Lapa MR. As clínicas populares como uma alternativa à saúde no Brasil: um estudo de caso em uma clínica popular. 2014. http://www.repositorio.ufc.br/handle/riufc/33277 (accessed May 8, 2020).

33 Gragnolati M, Lindelow M, Couttolenc B. Twenty Years of Health System Reform in Brazil. An Assessment of the Sistema Único de Saúde. Washington, DC: The World Bank, 2013.

34 Andrietta LS, Levi ML, Scheffer MC, Alves MTSS de B e, Oliveira BLCA de, Russo G. The differential impact of economic recessions on health systems in middle-income settings: a comparative case study of unequal states in Brazil. *BMJ Global Health* 2020; **5**: e002122.

35 MRC. How is the current crisis reshaping Brazil's health system? Strengthening health workforce and provision of services in São Paulo and Maranhão. UK Research and Innovation. 2018. https://gtr.ukri.org/projects?ref=MR%2FR022747%2F1 (accessed June 26, 2019).

36 Russo G, Cassenote A, De Oliveira BLCA, Scheffer M. Demographic and professional risk factors of SARS-CoV-2 infections among physicians in low- and middle-income settings: Findings from a representative survey in two Brazilian states. *PLOS Glob Public Health* 2022; **2**: e0000656.

37 Scheffer M, Cassenote A, de Britto E Alves MTSS, Russo G. The multiple uses of telemedicine during the pandemic: the evidence from a cross-sectional survey of medical doctors in Brazil. *Global Health* 2022; **18**: 81.

38 Dixon JC, Fullerton AS, Robertson DL. Cross-National Differences in Workers' Perceived Job, Labour Market, and Employment Insecurity in Europe: Empirical Tests and Theoretical Extensions†. *European Sociological Review* 2013; **29**: 1053–67.

39 Russo G, Levi ML, Seabra Soares de Britto E Alves MT, *et al.* How the 'plates' of a health system can shift, change and adjust during economic recessions: A

For peer review only - http://bmjopen.bmj.com/site/about/guidelines.xhtml

qualitative interview study of public and private health providers in Brazil's São Paulo and Maranhão states. *PLoS One* 2020; **15**: e0241017.

40 R Core Team — European Environment Agency. R: A language and environment for statistical computing. 2022. https://www.eea.europa.eu/data-and-maps/indicators/oxygen-consuming-substances-in-rivers/r-development-core-team-2006 (accessed March 9, 2022).

41 Cruz JAW, da Cunha MAVC, de Moraes TP, *et al.* Brazilian private health system: history, scenarios, and trends. *BMC Health Services Research* 2022; **22**: 49.

42 Miotto BA, Guilloux AGA, Cassenote AJF, Mainardi GM, Russo G, Scheffer MC. Physician's sociodemographic profile and distribution across public and private health care: an insight into physicians' dual practice in Brazil. *BMC Health Serv Res* 2018; **18**: 299.

43 Scheffer MC, Almeida C, Guilloux AGA, Guerra A, Miotto BA. Demografia médica no Brasil 2023. São Paulo (BRA): Faculdade de Medicina USP, Conselho Federal de Medicina, 2023.

44 Cockx B, Brasseur C. The demand for physician services. Evidence from a natural experiment. *J Health Econ* 2003; **22**: 881–913.

45 Baştav L. AN EMPIRICAL STUDY ON THE WAGE AND PRICE STICKINESS OF US ECONOMY (1990-2009) -. *Ekonomik Yaklasim* 2011; **22**: 45–64.

46 Haldane V, De Foo C, Abdalla SM, *et al.* Health systems resilience in managing the COVID-19 pandemic: lessons from 28 countries. *Nat Med* 2021; **27**: 964–80.

47 Williams OD. COVID-19 and Private Health: Market and Governance Failure. *Development* 2020; **63**: 181–90.

48 David Williams O, Yung KC, Grépin KA. The failure of private health services: COVID-19 induced crises in low- and middle-income country (LMIC) health systems. *Global Public Health* 2021; **16**: 1320–33.

49 Althubaiti A. Information bias in health research: definition, pitfalls, and adjustment methods. *J Multidiscip Healthc* 2016; **9**: 211–7.

50 Lee T, Propper C, Stoye G. Medical Labour Supply and the Production of Healthcare. *Fiscal Studies* 2019; **40**: 621–61.

For peer review only

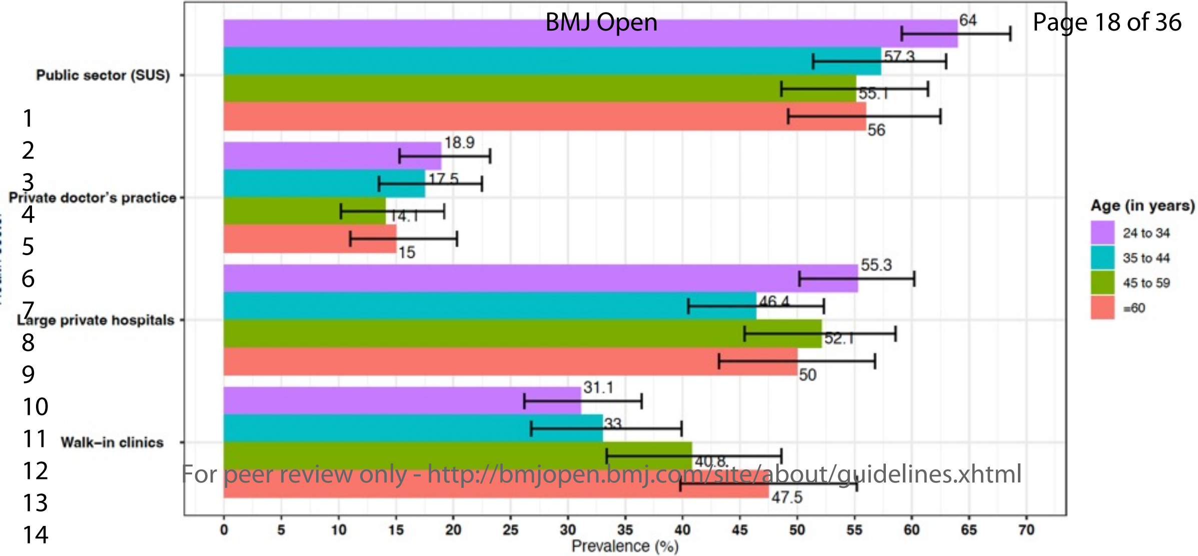

For peer review only - http://bmjopen.bmj.com/site/about/guidelines.xhtml

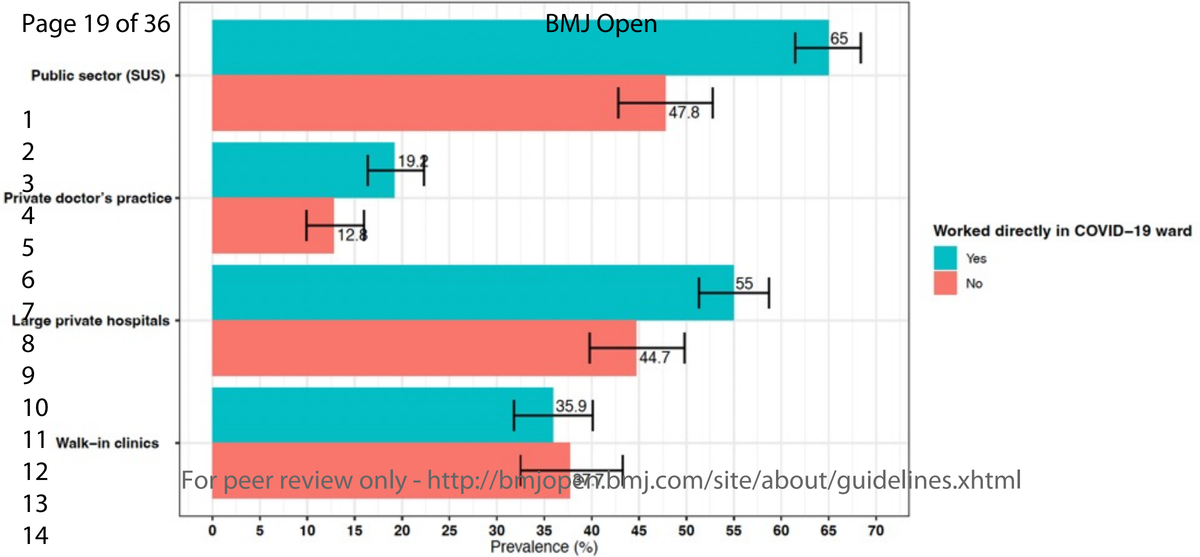

For peer review only - http://bmjopen.bmj.com/site/about/guidelines.xhtml

Title:
"What happened to health labour markets during COVID-19? An insight from a survey of medical doctors in Brazil"

Journal:
Applied Health Economics and Health Policy

Authors' information
Giuliano Russo (corresponding author), Wolfson Institute of Population Health, Queen Mary University of London, United Kingdom. Email: g.russo@qmul.ac.uk. ORCID ID: 0000-0002-2716-369X
Bruno Luciano Carneiro Alves de Oliveira, Program in Public Health, Federal University of Maranhão, São Luís 65080-805, Brazil. ORCID ID: 0000-0001-8053-7972
Alex Cassenote, Department of Preventive Medicine, University of São Paulo, Piracicaba 13416-000, Brazil. ORCID ID: 0000-0002-5098-1922
Mário Scheffer. Department of Preventive Medicine, University of São Paulo, Piracicaba 13416-000, Brazil. ORCID ID: 0000-0001-8931-6471

For peer review only

Datafolha INSTITUTO DE PESQUISAS

PM 745123 – SURVEY ON CORONAVIRUS IMPACT ON PHYSICIANS AND LABOUR MARKET IN SÃO PAULO AND MARANHÃO

					**CPD NO.**	
					**CITY NO.**	
**INSPECTION**	1. CHECKED	2. NO PHONE NUMBER	3. WRONG PHONE NUMBER	4. RESPONDENT NOT FOUND	**SPOT NO.**	
**CLOSED-ENDED QUESTIONS SCORE**				QTY:	**SURVEY NO.**	
**OPEN-ENDED QUESTIONS SCORE**				QTY:	**SURVEY TAKER NO.**	
**TYPE**	1. PROBABILISTIC	2. INTENTIONAL	3. ENLISTMENT		**HOUR START**	:
**INSPECTOR NO.**		**CRITIC NO.**		**DATE:** / /2020	**HOUR END**	:

Good morning/afternoon/evening. My name is ______. I work for Datafolha Research Institute and I am conducting a study, on behalf of ____ **(MENTION FROM LIST: IF SP > SAY USP; IF MA > SAY UFMA)** and the Federal Council of Medicine. Please, could I speak to Dr._______? **(PROCEED WITH THE NAME IN THE LIST. REPEAT FROM START IF THE CALL IS TRANSFERRED.)**

The survey we are conducting is about the medical work in Brazil within the context of the new coronavirus pandemic. The goal of this study is verifying the effects of the COVID-19 pandemic on the medical work in Brazil. This is an anonymous and confidential study that follows the standards of the Research Ethics Committee and the Medical Ethics Committee. Your name and answers will never be identified, and your data will remain stored only for the duration of the study. Do you agree to answer some questions?

1. Yes 2. No >> **END THE SURVEY**

For quality control purposes, this interview might be recorded.

GENDER 1. Male 2. Female

AGE What is your age? **(SPONTANEOUS ANSWER, ASK ONLY ONCE)**

[ ] **WRITE DOWN**

1. From 24 to 34 years
2. From 35 to 44 years
3. From 45 to 59 years
4. 60 years or older

QF.4 Do you currently work in the state of______ **(SÃO PAULO/ MARANHÃO – MENTION ACCORDING TO THE LIST)**

1. Yes
2. No **>> END THE SURVEY**

CRM:

[ ] **(GET FROM LIST)**

For peer review only - http://bmjopen.bmj.com/site/about/guidelines.xhtml

QF.3 In what year did you graduate? **(SPONTANEOUS ANSWER, ASK ONLY ONCE)**

(GET FROM LIST)

QF.1 Do you work as a physician or currently works with medicine or health?

1. Yes, full time (exclusivity contract) **PROCEED TO Q.36**
2. Yes, half time (partial dedication)
3. Does not work as physician / does not currently work with medicine or health

QF.2 (IF PF.1= 2 or 3) Did the decision of (giving partial dedication **IF QF1=2**) or (refrain from dedicating to medical work **IF PF1=3**) occur after March 2020, due to the pandemic?

1. Yes **PROCEED**
2. No **THANK THE RESPONDENT AND END THE SURVEY**

SET 1 – RESPONDENT CHARACTERIZATION

P.36 Have you been vaccinated, or do you intend to get vaccinated against COVID-19? **(SPONTANEOUS ANSWER, ASK ONLY ONCE)**

1. He/she is vaccinated
2. He/she has not been vaccinated, but intends to
3. He/she does not intend to get vaccinated

(SURVEY TAKER, READ THE FOLLOWING) THE COVID-19 PANDEMIC START IN BRAZIL IN MARCH 2020. I WILL ASK SOME QUESTIONS ABOUT YOUR WORK BEFORE AND AFTER THE START OF THE PANDEMIC.

P.1 Considering your current medical work, with its routine, format, volume of patients, and working hours, when compared to a usual level of work, before March 2020: **(READ PAUSEDLY UNTIL THE QUESTION MARK)**

1. It has not been impacted by the pandemic, performs the same work
2. Has been impacted, but resumed what used to be done before
3. Still suffers from impacts caused by the pandemic
4. The pandemic brought changes that will be permanently incorporated in his/her work
5. Physician work was interrupted due to the pandemic (is not currently working as a physician) **OR**
6. Retired from/definitively abandoned physician work due to the pandemic?

Q.2 Do you work, or did you work, on a regular basis, with medicine or health _______ ? **(READ EACH ITEM IN THE TABLE)** With any other area or type of activity that was not mentioned? **(IF YES, WRITE DOWN THE AREA OR ACTIVITY) (ENCOURAGED ANSWER AND SINGLE ANSWER PER LINE)**

For peer review only

Datafolha INSTITUTO DE PESQUISAS

Q.3 (FOR EACH Q.2=1) In this medical activity _____ **(READ THE ITEM)**, do you attend or did you attend, on a regular basis, only to patients with health insurance or private patients; attend/attended only SUS patients – (either at a public, philanthropic facility, Social Organization or another type of facility that provides services to SUS) OR attend /attended to both types of patients, work in both public and private sectors? **(ENCOURAGED ANSWER AND SINGLE ANSWER PER LINE)**

SHUFFLE ITEMS		Q.2 TYPE OF MEDICAL PRACTICE		Q.3 SECTOR OF WORK		
		YES	NO	PUBLIC	PRIVATE	BOTH
a.	With appointments at the office, ward or clinic?	1	2	1	2	3
b.	In the handling of diagnostic testing equipment?	1	2	1	2	3
c.	With surgeries in a hospital that require hospitalization and general/epidural/spinal anesthesia?	1	2	1	2	3
d.	With ward surgeries, without hospitalization and local anesthesia?	1	2	1	2	3
e.	Management, direction, service administration and institutional roles?	1	2	1	2	3
f.	Teaching or research roles?	1	2	1	2	3
ALWAYS ASK AS LAST QUESTION						
g.	Do you work in another sector or type of activity that was not mentioned? What___?	1	2	1	2	3

Q.4 (IF Q.3=2 or 3 IN AT LEAST ONE ITEM) You said that you work/worked in the private sector, attending to patients with health insurance or private patients. I will read a list of some workplaces in the private sector, and I would like to know in each one of them you work at or used to work at on a regular basis.

Do you work or did you work at _______ **(READ EACH ONE OF THE ITEMS)**? Is there any other place of the private sector that I have not mentioned? **(ENCOURAGED ANSWER AND SINGLE ANSWER PER ITEM)**

Q.5 (FOR EACH Q4=1) Approximately how many hours do you currently work at ____ **(READ EACH ITEM)** per week? **(ENCOURAGED ANSWER AND SINGLE ANSWER PER ITEM)**

SHUFFLE ITEMS	P.4 PRIVATE SECTOR WORK		P.5 HOURS/ WEEK
	YES	NO	(WRITE DOWN THE HOURS)

				9996. Zero hours
a.	Own private office or shared with one or more colleagues?	1	2	______
b.	Private clinic or ward, where you provide care and services?	1	2	______
c.	Affordable private clinics<br><br>*(Example: Dr. Consulta, Cia da Consulta, Doutor Hoje for São Paulo; or Super Clínica, Mega Clínica, Super Clínica, Dr. Saúde for Maranhão)*	1	2	______
e.	Private hospital?	1	2	______
f.	Private diagnostic laboratory or private clinical analysis facility?	1	2	______
g.	Pharmaceutical industry?	1	2	______
h.	Medical sector of a company?	1	2	______
i.	Private university?	1	2	______
**ALWAYS ASK AS LAST QUESTION**				
k.	Is there any other place of the private sector that I have not mentioned? **(IF YES)** What? ___________ **(WRITE DOWN)**	1	2	______

Q.6 (IF Q.3=1 OR 3 IN AT LEAST ONE ITEM) You said that you work/worked in the public sector, attending to SUS patients or in public services or institutions. I will read a list of some workplaces in the public sector, and I would like to know in which one of them do you work at or used to work at on a regular basis.

Do you work or did you work at _______ **(READ EACH ONE OF THE ITEMS)**? Is there any other place of the private sector that I have not mentioned? **(ENCOURAGED ANSWER AND SINGLE ANSWER PER ITEM)**

Q.7 (FOR EACH Q.6=1) Approximately how many hours do you currently work at _____ **(READ EACH ITEM)** per week? **(ENCOURAGED ANSWER AND SINGLE ANSWER PER ITEM)**

SHUFFLE ITEMS	Q.6 PUBLIC SECTOR WORK		P.7 HOURS/WEEK
	YES	NO	(WRITE DOWN HOURS)<br><br>9996. Zero hours

For peer review only - http://bmjopen.bmj.com/site/about/guidelines.xhtml

**a.**	Primary health care, such as: *Basic Health Unit, Health Center, Family Health Program, Family Health Strategy*	**1**	**2**	______
**b.**	Ward services, such as: *Specialty Ward, AMA, mental health services – CAPs; HIV-Aids services; Hemocentre and Hemotherapy; Occupational Health Services*	**1**	**2**	______
**c.**	Urgent and emergency services, such as: *First aid services, emergency care, UPA (Emergency Unit), rescue, SAMU (Urgent Medical Assistance Service), pre-hospital care*	**1**	**2**	______
**d.**	Public hospital, such as: *SUS Hospital or an affiliated hospital that attends to SUS patients* *(Municipal, State or Federal Hospitals, University Hospitals, Charity Hospitals, Philanthropic Hospitals, Hospitals managed by Social Organization, Foundation or Hospital Authority)*	**1**	**2**	______
**g.**	Public university or public research institutions?	**1**	**2**	______
**h.**	Administrative or management services of public nature	**1**	**2**	______
**ALWAYS ASK AS LAST QUESTION**				
**k.**	Is there any other place of the public sector that I have not mentioned? **(IF YES)** What? __________ **(WRITE DOWN)**	**1**	**2**	______

ASK TO ALL RESPONDENTS

Q.8 I will read now some different regimes of employment or provision of medical services and I would like to know in which one of them you currently find yourself ______ **(ENCOURAGED ANSWER AND SINGLE ANSWER PER ITEM)**

Q.9 Before the pandemic, before March 2020, how was your employment regime ______ **(READ EACH ITEM)**?

**SHUFFLE ITEMS**		**Q.8 CURRENT EMPLOYMENT METHOD**		**Q.9 EMPLOYMENT METHOD BEFORE THE PANDEMIC**	
		**YES**	**NO**	**YES**	**NO**
**A.**	Statutory, civil servant?	1	2	1	2
**B**	CLT employment?	1	2	1	2
**C.**	Legal entity – PJ?	1	2	1	2
**D.**	Partner in a legal entity with other professionals?	1	2	1	2
**E.**	Cooperative?	1	2	1	2
**F.**	Freelancer with Self-employment income receipt?	1	2	1	2
**G.**	Contracts or individual agreements of another nature?	1	2	1	2

For peer review only - http://bmjopen.bmj.com/site/about/guidelines.xhtml

Q.10 I will read some income ranges and I would like to know in what range were you in before the pandemic, before March 2020. Considering all your works and employments related to the practice of medicine that you had, approximately how much did you earn per month? **(ENCOURAGED ANSWER AND SINGLE ANSWER)**

1. Up to R$6,000
2. From R$6,001 to R$ 11,000
3. From R$ 11,001 to R$ 16,000
4. From R$ 16,001 to R$ 21,000
5. From R$ 21,001 to R$ 27,000
6. From R$ 27,001 to R$ 32,000
7. From R$ 32,001 to R$ 40,000
8. R$40,001 or more

9997. Refused to answer **(SPONTANEOUS ANSWER)**

SET II: CHANGES IN WORK OF RESPONDENTS CAUSED BY COVID-19 (both public and private sectors)

Q.11. Since March 2020, have you worked in activities/services directly related to COVID-19 by attending to infected/sick patients or in activities against the pandemic? **(IF YES)** Are you currently working in activities related to COVID-19? **(SPONTANEOUS AND SINGLE ANSWER)**

1. Have not worked and neither is working in any activity directly related to COVID-19.
   IF YES:
2. Is currently working in activities related to COVID-19.
3. Has worked in activities directly related to COVID-19 but is no longer working at the moment.

Q.12. (IF Q.11 = 2 or 3) In what kind of work, activity or place related to COVID-19 did you work/have you worked? **(ENCOURAGED ANSWER AND SINGLE ANSWER PER ITEM)**

**SHUFFLE ITEMS**		**Q.12**	
		**YES**	**NO**
**a.**	In hospital care of patients with COVID in ward or ICU	**1**	**2**
**b.**	In the care of suspicious or confirmed cases that do not require hospitalization	**1**	**2**
**c.**	In teleconsultation or other non-presential guidance or assistance activities related to COVID	**1**	**2**
**d.**	In scientific/academic research on COVID	**1**	**2**
**g.**	In health surveillance, committees or other governmental actions related to COVIC	**1**	**2**
**ALWAYS ASK AS LAST QUESTION**			
**h.**	Any other activity? What? ______ **(WRITE DOWN)**	**1**	**2**

For peer review only

Datafolha INSTITUTO DE PESQUISAS

Q.13 Considering a regular/habitual work week before the start of the pandemic, and your work after March 2020, has your number of worked hours per week increased, remained the same or decreased? **(ENCOURAGED ANSWER AND SINGLE ANSWER PER ITEM)**

1. Increased
2. Remained the same
3. Decreased

Q.14a (IF Q13=1) Approximately in how many weekly hours would you say that your work has increased since the start of the pandemic? **(SPONTANEOUS AND SINGLE ANSWER)**

________ hours **(WRITE DOWN)** **9999.** Does not know

Q.14b (IF Q13=3) Approximately in how many weekly hours would you say that your work has decreased since the start of the pandemic? **(SPONTANEOUS AND SINGLE ANSWER)**

________horas **(WRITE DOWN)** **9999.** Does not know

Q.15 Considering your monthly salary before March 2020 – at the start of the pandemic, has your salary been increased, remained the same or decreased? (**ENCOURAGED AND SINGLE ANSWER)**

1. Increased
2. Remained the same
3. Decreased

Q.16a (IF Q.15 = 1) How much, in Reais, has your monthly salary been increased? **(ENCOURAGED AND SINGLE ANSWER)**

(WRITE DOWN)

R$

9997. Refuses to answer
9999. Does not know

P.16b (SE P.15= 3) How much, in Reais, has your monthly salary been decreased? **(ENCOURAGED AND SINGLE ANSWER)**

(WRITE DOWN)

R$

9997. Refuses to answer
9999. Does not know

P.18 During the pandemic, how much do you think would be a fair amount for one hour of overtime work, in Reais? **(SPONTANEOUS AND SINGLE ANSWER)**

(WRITE DOWN)

R$

9997. Refuses to answer
9999. Does not know

For peer review only

Q.19 Were you diagnosed with COVID-19 at any moment? **(IF YES)** Were you asymptomatic, with mild symptoms or did you require hospitalization and/or ICU? **(ENCOURAGED AND SINGLE ANSWER)**

1. Yes, I was asymptomatic
2. Yes, I had mild symptoms
3. Yes, I required hospitalization and/or ICU.
4. I have not been diagnosed with COVID-19.

Q.20 (IF Q.19 ≠ 4) How many working days did you miss due to the COVID-19 diagnosis, up until now? **(SPONTANEOUS AND SINGLE ANSWER)**

**(WRITE DOWN)**

9996. None
9999. Does not know

Q.21. Do you_______ **(READ EACH ITEM)? (ENCOURAGED AND SINGLE ANSWER)**

**SHUFFLE ITEMS**		**Q.22**	
		**YES**	**NO**
**a.**	Carry out appointments and guidance to patients by telemedicine?	**1**	**1**
**b.**	Have work meetings by telemedicine?	**1**	**2**
**c.**	Have case discussions with colleagues by telemedicine?	**1**	**2**
**d.**	Perform prescriptions, certificates or reports by remote methods or telemedicine	**1**	**2**
**g.**	Do you prepare/annotate electronic medical records by telemedicine?	**1**	**2**
**h.**	Receive medical qualification or training by telemedicine	**1**	**2**
**ALWAYS ASK AS LAST QUESTION**			
**i.**	Perform any other activity by telemedicine? What? _____ **(WRITE DOWN)**	**1**	**2**

Q.22 (IF Q.21=1 IN AT LEAST OE ITEM) Considering your relationship with telemedicine/remote consultation, you: **(READ UNTIL THE QUESTION MARK) (ENCOURAGED AND SINGLE ANSWER)**

1. Had already been using this resource before the pandemic and kept using them,
2. Had never used this resource, but started using it due to the pandemic **OR**
3. Used the resource before, but no longer uses it since the beginning of the pandemic?
4. Had already used telemedicine occasionally to receive qualification, but has never used it with patients (SPONTANEOUS ANSWER)

For peer review only

Q.23 (IF Q.22 = 1 or 2) Currently, in your professional activities, how many hours do you dedicate yourself on a weekly basis to digital platforms/telemedicine/remote consultation? **(SPONTANEOUS AND SINGLE ANSWER)**

(ATTENTION, SURVEY TAKER, USE THE BOX WITH MINUTES ONLY IF THE RESPONDENT SPONTANEOUSLY SAYS THE TIME IN MINUTES AND IF HE/SHE MENTIONS LESS THAN 60 MINUTES)

**(WRITE DOWN in hours)**
**(WRITE DOWN in minutes)**<br><br>**(SPONTANEOUS ANSWER)**

9999. Does not know

SET III: CHANGES IN THE MEDICAL LABOR MARKET

Q.24 In your opinion, comparing to before the pandemic, at SUS____ **(READ UNTIL THE QUESTION MARK – ENCOURAGED AND SINGLE ANSWER)**

1. There are fewer work opportunities
2. There have been no significant changes in work opportunities **OR**
3. There are more work opportunities**?**

9999. Does not know

Q.25a (IF Q.24=3) Using a score from one to ten, how much do you think that the work opportunities at SUS have been increased when compared to before the start of the pandemic?

ZERO										TEN	DOES NOT KNOW
9996	1	2	3	4	5	6	7	8	9	10	9999

Q.25b (IF Q.24=1) Using a score from one to ten, how much do you think that the work opportunities at SUS have been decreased when compared to before the start of the pandemic?

ZERO										TEN	DOES NOT KNOW
9996	1	2	3	4	5	6	7	8	9	10	9999

For peer review only

Q.26 In your opinion, comparing to before the start of the pandemic, at the private offices _____ **(READ UNTIL THE QUESTION MARK – ENCOURAGED AND SINGLE ANSWER)**

1. There are fewer work opportunities
2. There have been no significant changes in work opportunities **OR**
3. There are more work opportunities**?**

9999. Does not know

Q.27a (IF Q.26=3) Using a score from one to ten, how much do you think that the work opportunities at private offices have been increased when compared to before the start of the pandemic?

ZERO										TEM	DOES NOT KNOW
9996	1	2	3	4	5	6	7	8	9	10	9999

Q.27b (IF Q.27=1) Using a score from one to ten, how much do you think that the work opportunities at private offices have been decreased when compared to before the start of the pandemic?

ZERO										TEN	DOES NOT KNOW
9996	1	2	3	4	5	6	7	8	9	10	9999

Q.28 In your opinion, comparing to before the start of the pandemic, at the private hospitals _____ **(READ UNTIL THE QUESTION MARK – ENCOURAGED AND SINGLE ANSWER)**

1. There are fewer work opportunities
2. There have been no significant changes in work opportunities **OR**
3. There are more work opportunities**?**

9999. Does not know

Q.29a (IF Q.28=3) Using a score from one to ten, how much do you think that the work opportunities at private hospitals have been increased when compared to before the start of the pandemic?

ZERO										TEN	DOES NOT KNOW
9996	1	2	3	4	5	6	7	8	9	10	9999

Q.29b (IF Q.28=1) Using a score from one to ten, how much do you think that the work opportunities at private hospitals have been decreased when compared to before the start of the pandemic?

For peer review only - http://bmjopen.bmj.com/site/about/guidelines.xhtml

Datafolha INSTITUTO DE PESQUISAS

ZERO										TEN	DOES NOT KNOW
9996	1	2	3	4	5	6	7	8	9	10	9999

Q.30 In your opinion, comparing to before the start of the pandemic, at the affordable private clinics _____ **(READ UNTIL THE QUESTION MARK – ENCOURAGED AND SINGLE ANSWER)**

1. There are fewer work opportunities
2. There have been no significant changes in work opportunities **OR**
3. There are more work opportunities**?**

9999. Does not know

Q.31a (IF Q.30=3) Using a score from one to ten, how much do you think that the work opportunities at affordable private clinics have been increased when compared to before the start of the pandemic?

ZERO										TEN	DOES NOT KNOW
9996	1	2	3	4	5	6	7	8	9	10	9999

Q.31b (IF Q.30=1) Using a score from one to ten, how much do you think that the work opportunities at affordable private clinics have been decreased when compared to before the start of the pandemic?

ZERO										TEN	DOES NOT KNOW
9996	1	2	3	4	5	6	7	8	9	10	9999

Q.32 In your opinion, comparing to before the pandemic, the availability of physicians for hiring ________ **(READ UNTIL THE QUESTION MARK – ENCOURAGED AND SINGLE ANSWER)**

1. Has been decreased
2. There have been no significant changes in the number of medical professionals available **OR**
3. Has been increased**?**

9999. Does not know

Q.33a (IF Q.32=3) Using a score from one to ten, how much do you think that the availability of medical professionals for hiring has been increased, when compared to before the start of the pandemic?

ZERO										TEN	DOES NOT KNOW

For peer review only - http://bmjopen.bmj.com/site/about/guidelines.xhtml

9996	1	2	3	4	5	6	7	8	9	10	9999

Q.33b (IF Q.32=1) Using a score from one to ten, how much do you think that the availability of medical professionals for hiring has been decreased, when compared to before the start of the pandemic?

ZERO										TEN	DOES NOT KNOW
9996	1	2	3	4	5	6	7	8	9	10	9999

Q.34 In your opinion, comparing to before the pandemic, the amount paid to physicians on duty in your specialty ________ **(READ UNTIL THE QUESTION MARK – ENCOURAGED AND SINGLE ANSWER)**

1. Has been decreased
2. There have been no significant changes in the amount paid to physicians in your specialty **OR**
3. Has been increased**?**

9999. Does not know

Q.35a (IF Q34=3) In terms of a percentage, how much do you think that the amount paid to physicians on duty in your specialty has been increased, when compared to before the pandemic? **(SINGLE ANSWER)**

**(WRITE DOWN – 0% to 100%)**

9999. Does not know

Q.35b (IF Q34=1) In terms of a percentage, how much do you think that the amount paid to physicians on duty in your specialty has been decreased, when compared to before the pandemic? **(SINGLE ANSWER)**

**(WRITE DOWN– 0% to 100%)**

9999. Does not know

I declare hereby that:
1. this survey was conducted in accordance with the instructions of the field supervision;
2. the information in this survey were correctly written down and they accurately correspond to the declarations of the respondent;
3. I am aware that the material collected by me is being or will be checked for quality control purposes;
4. I am aware that I must keep the confidentiality of the collected information;
5. I shall not reproduce this survey nor the information in it for my own use or third-party use.

NAME : ____

For peer review only

Datafolha
INSTITUTO DE PESQUISAS

SIGNATURE: ____________________________ ID. Doc. No.: ___________________ Date: ____/ ___ / ___

For peer review only

Title:

"What happened to health labour markets during COVID-19? An insight from a survey of medical doctors in Brazil"

Journal:

Applied Health Economics and Health Policy

Authors' information

Giuliano Russo (corresponding author), Wolfson Institute of Population Health, Queen Mary University of London, United Kingdom. Email: g.russo@qmul.ac.uk. ORCID ID: 0000-0002-2716-369X

Bruno Luciano Carneiro Alves de Oliveira, Program in Public Health, Federal University of Maranhão, São Luís 65080-805, Brazil. ORCID ID: 0000-0001-8053-7972

Alex Cassenote, Department of Preventive Medicine, University of São Paulo, Piracicaba 13416-000, Brazil. ORCID ID: 0000-0002-5098-1922

Mário Scheffer. Department of Preventive Medicine, University of São Paulo, Piracicaba 13416-000, Brazil. ORCID ID: 0000-0001-8931-6471

For peer review only

Table 1: Physicians sample, but location and socio-economic characteristics

Physicians' sociodemographic characteristics	Proportion of total (n = 1,183)	São Paulo (n = 632) 53.4%	Maranhão (n = 551) 46.6%
Gender			
Male	56.2	54.1	58.6
Female	43.8	45.9	41.4
Age			
24 to 34	34.1	34.3	33.9
35 to 44	24.5	20.7	28.9
45 to 59	20.4	22.3	18.1
≥60	21.0	22.6	19.1
Geographical location of deployment			
Rural areas (Interior)	50.5	54.9	45.6
Urban areas around capital cities	49.5	45.1	54.5
Health sector of deployment			
Exclusively public	19.6	14.2	25.8
Exclusively private	22.1	31.5	11.3
Dual practice	58.3	54.3	63.0
Working directly with COVID-19 patients			
Yes	63.4	59.2	68.2
No	36.6	40.8	31.8

Table 2: Physicians' perceptions on employment opportunities in public and private facilities, by State

Employment opportunities		Total (n=1,181)		São Paulo (n=632) 53.4% (50.6-56.3)		Maranhão (n=551) 46.6% (43.7-49.4)	
		%	IC95%	%	IC95%	%	IC95%
**Public sector**							
SUS	Increased	72.2	(66.1-77.7)	67.4	(57.0-76.4)	75.2	(67.4-81.6)
	Reduced	11.9	(8.3-16.8)	18.6	(11.8-28.1)	7.8	(4.4-13.4)
	No changes	15.9	(11.7-21.2)	14.0	(8.2-22.8)	17.0	(11.7-24.1)
Private doctor's practice	Increased	18.8	(13.9-25.0)	13.0	(7.0-23.0)	22.2	(15.6-30.6)
	Reduced	55.4	(48.2-62.3)	65.2	(53.4-75.4)	49.6	(40.7-58.5)
	No changes	25.8	(20.1-32.5)	21.7	(13.6-32.8)	28.2	(20.8-37.0)
Large private hospitals	Increased	70.2	(63.5-76.1)	68.9	(57.7-78.3)	71.0	(62.4-78.2)
	Reduced	12.6	(8.7-18.0)	14.9	(8.5-24.7)	11.3	(6.9-18.1)

For peer review only

	No changes	17.2	(12.6-23.0)	16.2	(9.5-26.2)	17.7	(12.0-25.4)
Walk-in private clinics	Increased	40.9	(34.0-48.2)	45.9	(34.0-58.3)	38.3	(30.1-47.3)
	Reduced	21.0	(15.7-27.5)	21.3	(12.9-33.1)	20.8	(14.5-28.9)
	No changes	38.1	(31.4-45.4)	32.8	(22.3-45.3)	40.8	(32.5-49.8)
**Private sector**							
SUS	Increased	53.1	(46.4-59.6)	47.4	(39.7-55.3)	67.8	(55.1-78.3)
	Reduced	17.8	(13.3-23.5)	19.5	(14.0-26.4)	13.6	(7.0-24.5)
	No changes	29.1	(23.4-35.5)	33.1	(26.2-40.9)	18.6	(10.7-30.4)
Private doctor's practice	Increased	12.2	(8.7-16.7)	9.2	(5.9-14.0)	22.0	(13.4-34.1)
	Reduced	62.7	(56.7-68.4)	65.8	(58.9-72.1)	52.5	(40.0-64.7)
	No changes	25.1	(20.2-30.8)	25.0	(19.5-31.5)	25.4	(16.1-37.8)
Large private hospitals	Increased	49.6	(43.3-55.9)	45.2	(38.0-52.6)	62.7	(50.0-73.9)
	Reduced	24.2	(19.1-30.0)	26.0	(20.1-32.9)	18.6	(10.7-30.4)
	No changes	26.3	(21.1-32.2)	28.8	(22.6-35.9)	18.6	(10.7-30.4)
Walk-in private clinics	Increased	37.1	(30.3-44.5)	36.4	(28.6-45.0)	39.1	(26.4-53.5)
	Reduced	28.6	(22.4-35.7)	29.5	(22.3-37.8)	26.1	(15.6-40.3)
	No changes	34.3	(27.7-41.6)	34.1	(26.5-42.6)	34.8	(22.7-49.2)
**Dual practice**							
SUS	Increased	56.4	(52.6-60.1)	49.8	(44.4-55.3)	62.5	(57.3-67.5)
	Reduced	18.0	(15.3-21.1)	21.8	(17.6-26.6)	14.5	(11.1-18.6)
	No changes	25.6	(22.4-29.1)	28.3	(23.7-33.5)	23.0	(18.8-27.8)
Private doctor's practice	Increased	18.1	(15.4-21.2)	16.6	(12.9-21.0)	19.6	(15.7-24.2)
	Reduced	62.5	(58.8-66.1)	64.7	(59.4-69.7)	60.4	(55.1-24.5)
	No changes	19.3	(16.5-22.5)	18.7	(14.9-23.3)	19.9	(16.0-24.5)
Large private hospitals	Increased	46.3	(42.4-50.1)	43.4	(38.0-48.9)	49.1	(43.7-54.5)
	Reduced	32.2	(28.7-36.0)	36.1	(31.0-41.5)	28.5	(23.9-33.7)
	No changes	21.5	(18.5-24.8)	20.6	(16.5-25.4)	22.4	(18.2-27.2)
Walk-in private clinics	Increased	34.6	(30.4-39.1)	40.1	(33.6-47.0)	30.4	(25.1-36.2)
	Reduced	31.8	(27.7-36.2)	27.7	(22.0-34.3)	35.0	(29.5-41.0)
	No changes	33.5	(29.4-38.0)	32.2	(26.1-38.9)	34.6	(29.1-40.6)
**Total**							
SUS	Increased	59.0	(56.1-61.9)	51.9	(47.7-56.0)	66.4	(62.3-70.3)
	Reduced	16.7	(14.6-19.0)	20.7	(17.5-24.2)	12.6	(10.1-15.7)
	No changes	24.3	(21.8-26.9)	27.5	(23.9-31.3)	21.0	(17.7-24.6)
Private doctor's practice	Increased	15.8	(13.8-18.0)	13.7	(11.2-16.7)	20.5	(17.2-24.2)
	Reduced	57.4	(54.5-60.2)	65.1	(61.2-68.9)	57.0	(52.7-61.3)
	No changes	20.3	(18.1-22.7)	21.2	(18.0-24.6)	22.5	(19.1-26.3)

For peer review only

Hospitais Large private hospitals	Increased	51.4	(48.4-54.4)	47.3	(43.2-51.4)	56.0	(51.7-60.2)
	Reduced	26.9	(24.3-29.6)	30.2	(26.5-34.1)	23.2	(19.7-27.0)
	No changes	21.7	(19.4-24.3)	22.6	(19.3-26.2)	28.8	(17.5-24.6)
Walk-in private clinics	Increased	36.6	(33.3-39.9)	39.8	(35.1-44.7)	33.6	(29.2-38.2)
	Reduced	28.7	(25.7-31.9)	27.3	(23.1-31.9)	30.0	(25.9-34.6)
	No changes	34.7	(31.5-38.0)	32.9	(28.4-37.7)	36.4	(32.0-41.1)

Source: USP-UFMA-QMUL (2022)

Table 3: Perceptions on changes in remuneration for 12h A&E shift

Remuneration per shift		Total (n=1,181)		São Paulo (n=632) 53.4% (50.6-56.3)		Maranhão (n=551) 46.6% (43.7-49.4)	
		%	IC95%	%	IC95%	%	IC95%
Público	Increased	15.7	(11.5-21.2)	14.8	(8.7-24.1)	16.3	(11.0-23.4)
	Reduced	7.9	(5.0-12.2)	7.4	(3.4-15.2)	8.2	(4.6-14.0)
	No changes	76.4	(70.3-81.6)	77.8	(67.7-85.5)	75.6	(67.7-82.0)
Privado	Increased	8.6	(5.5-13.2)	8.8	(5.3-14.2)	8.0	(3.2-18.8)
	Reduced	10.5	(7.0-15.4)	10.7	(6.8-16.5)	10.0	(4.4-21.4)
	No changes	80.9	(75.0-85.6)	80.5	(73.7-85.9)	82.0	(69.2-90.2)
Dupla prática	Increased	12.4	(10.0-15.1)	12.8	(9.6-16.9)	11.9	(8.8-16.0)
	Reduced	14.9	(12.3-17.8)	16.2	(12.6-20.6)	13.5	(10.2-17.7)
	No changes	72.8	(69.2-76.1)	71.0	(65.8-75.7)	74.5	(69.5-79.0)
Total	Increased	12.3	(10.5-14.4)	11.9	(9.5-14.9)	12.7	(10.1-15.9)
	Reduced	12.6	(10.7-14.7)	13.4	(10.8-16.4)	11.7	(9.2-14.8)
	No changes	75.1	(72.4-77.6)	74.7	(70.9-78.1)	75.5	(71.6-79.1)

Source: USP-UFMA-QMUL (2022)

For peer review only

BMJ Open

What happened to health labour markets during COVID-19? Insights from the analysis of cross-sectional survey data on the perceptions of medical doctors in Brazil

Journal:	*BMJ Open*
Manuscript ID	bmjopen-2023-075458.R1
Article Type:	Original research
Date Submitted by the Author:	06-Jul-2023
Complete List of Authors:	Russo, Giuliano; Queen Mary University of London, Wolfson Institute of Population Health<br>de Oliveira, Bruno Luciano Carneiro Alves; Universidade Federal do Maranhao, Curso de Medicina<br>Cassenote, Alex; Universidade de São Paulo Hospital das Clínicas<br>Scheffer, Mário; Universidade de Sao Paulo Faculdade de Medicina, Preventative Medicine
**Primary Subject Heading**:	Global health
Secondary Subject Heading:	Health economics, Health policy, Health services research
Keywords:	COVID-19, Health economics < HEALTH SERVICES ADMINISTRATION & MANAGEMENT, International health services < HEALTH SERVICES ADMINISTRATION & MANAGEMENT, Physicians, Surveys and Questionnaires, Human resource management < HEALTH SERVICES ADMINISTRATION & MANAGEMENT

SCHOLARONE™ Manuscripts

I, the Submitting Author has the right to grant and does grant on behalf of all authors of the Work (as defined in the below author licence), an exclusive licence and/or a non-exclusive licence for contributions from authors who are: i) UK Crown employees; ii) where BMJ has agreed a CC-BY licence shall apply, and/or iii) in accordance with the terms applicable for US Federal Government officers or employees acting as part of their official duties; on a worldwide, perpetual, irrevocable, royalty-free basis to BMJ Publishing Group Ltd ("BMJ") its licensees and where the relevant Journal is co-owned by BMJ to the co-owners of the Journal, to publish the Work in this journal and any other BMJ products and to exploit all rights, as set out in our licence.

The Submitting Author accepts and understands that any supply made under these terms is made by BMJ to the Submitting Author unless you are acting as an employee on behalf of your employer or a postgraduate student of an affiliated institution which is paying any applicable article publishing charge ("APC") for Open Access articles. Where the Submitting Author wishes to make the Work available on an Open Access basis (and intends to pay the relevant APC), the terms of reuse of such Open Access shall be governed by a Creative Commons licence – details of these licences and which Creative Commons licence will apply to this Work are set out in our licence referred to above.

Other than as permitted in any relevant BMJ Author's Self Archiving Policies, I confirm this Work has not been accepted for publication elsewhere, is not being considered for publication elsewhere and does not duplicate material already published. I confirm all authors consent to publication of this Work and authorise the granting of this licence.

For peer review only

TITLE PAGE

Title: What happened to health labour markets during COVID-19? Insights from the analysis of cross-sectional survey data on the perceptions of medical doctors in Brazil

Running heading: Health labour market in Brazil during COVID-19

AUTHORS' INFORMATION

Giuliano Russo (corresponding author), Wolfson Institute of Population Health, Queen Mary University of London, United Kingdom. Email: g.russo@qmul.ac.uk. ORCID ID: 0000-0002-2716-369X

Bruno Luciano Carneiro Alves de Oliveira, Program in Public Health, Federal University of Maranhão, São Luís 65080-805, Brazil. ORCID ID: 0000-0001-8053-7972.

Alex Cassenote, Department of Preventive Medicine, University of São Paulo, Piracicaba 13416-000, Brazil. ORCID ID: 0000-0002-5098-1922.

Mário Scheffer. Department of Preventive Medicine, University of São Paulo, Piracicaba 13416-000, Brazil. ORCID ID: 0000-0001-8931-6471.

ABSTRACT

Objectives. To examine physicians' perceptions of changing employment opportunities in Brazil, and gain an insight into labour markets in low- and middle-income countries (LMICs) during the pandemic.

Study design. Descriptive and inferential analysis of a quantitative dataset from a representative cross-sectional survey of physicians of two Brazilian states.

Settings. São Paulo and Maranhão states in Brazil.

Participants. Representative sample of 1,183 physicians

Outcome measures. We estimated prevalence and 95% Confidence Intervals (CI) for physicians' perceptions of changes in demand and supply of doctors, as well as changes in prices of medical services for facilities of practice in the two States, stratified by public, private, and dual practice physicians.

Results. Most doctors reported increased job opportunities in the public sector (54.9%, 95% CI 52.0-57.7), particularly in Maranhão state (65.0%, 95% CI 60.9-68.9). For the private sector, increased opportunities were reported only in large private hospitals (46.7%, 95% CI 43.9-49.6), but not in smaller clinics. We recorded perceptions of slight increases in availability of doctors in Maranhão, particularly in the public sector (51.4%, 95 CI 43.2-59.5). Younger doctors recounted increased vacancies in the public sector (64%, 95 CI 58.1-68.1); older doctors only in walk-in clinics in Maranhão (47.5%, 95 CI 39.9-55.1). Those working directly with COVID-19 saw opportunities in public hospitals (65%, 95 CI 62.3-68.4), and in large private ones (55%, 95 CI 51.8-59.1).

Conclusions. Our findings hint that health labour markets in LMICs may not necessarily shrink during epidemics, and that impacts will depend on the balance of public and private services in national health systems.

For peer review only - http://bmjopen.bmj.com/site/about/guidelines.xhtml

Strengths and limitations of this study

- This paper is part of a wider research project on the impact of COVID-19 on physicians
- We employ a dataset from a representative sample of physicians from two Brazilian States
- We used labour market theory to interpret changes in availability of doctors during epidemics
- However, our analysis is based on physicians' perceptions, which may not be objective measures of changes in labour markets
- São Paulo and Maranhão states may not be entirely representative of wider Brazil or other LMICs

Statements and Declarations

Competing Interests: The authors declare no competing interests.

Acknowledgments

This study on whose findings this paper is based, received support from the Confap-MRC call for Health Systems Research Networks. Specifically, Bruno de Oliveira was funded by the Fundação de Pesquisa do Estado Maranhão (FAPEMA, Processo COOPI-00709/18) and Coordenação de Aperfeiçoamento de Pessoal de Nível Superior-Brasil (CAPES)—Finance Code 001, to the Graduate Program in Public Health. Giuliano Russo received an award from the NEWTON FUND, MEDICAL RESEARCH COUNCIL (UK), grant reference MR/R022747/1. Mário Scheffer and Alex Cassenote received an award from the Fundação de Amparo à Pesquisa do Estado de São Paulo (FAPESP-Brazil), 2017/50356-7. The funders had no role in the survey's design, implementation, or analysis.

For peer review only

INTRODUCTION AND BACKGROUND

The COVID-19 pandemic and the ensuing economic crisis has had an unparalleled impact on population health, (1) national economies, (2) and working modalities.(3) A recent OECD report presents evidence on the ways labour markets have changed due to pandemic-related lockdown measures, as businesses were disrupted and workers furloughed or laid off.(4)

The economic literature has so far suggested that health labour markets are 'recession proof', that is, one of the few sectors to hold up during economic slowdowns (5). Scholars have argued this may be due to an inelastic demand for healthcare services, and to increasing health needs during recessions.(6) Evidence from the US in fact showed increasing employment opportunities in the healthcare industry during the 2008 financial crisis, particularly for nurses.(7) Studies from Canada and Australia concluded that labour markets for physicians were largely unaffected during the ensuing recession.(8,9) However, in those countries with publicly funded health systems, government-driven austerity measures did reduce health sector resources and shrink health labour markets, particularly in Europe.(10–12)

There is a growing body of literature highlighting the role of labour markets in the provision of healthcare services in both high-income countries and LMICs,(13,14) which tends to advocate for the use of Health Labour Market Analysis to design effective health policies in such contexts.(15,16)

During COVID-19, research has shown that global labour health markets experienced a multi-layered crisis amplified by the combination of lockdown measures and reduced worker mobility.(17) In the first year of the pandemic, studies from the US argued that health sector unemployment rose because of pay cuts and redundancies, as patients were delaying seeking treatment, and hospitals were focussing on COVID-19 care, which "is not where the money is".(18) Other US scholars (19) noticed that unemployment in the healthcare industry increased less than in other sectors during COVID-19; while less specialised jobs (such as non-healthcare hospital workers and therapists) were badly affected, employment opportunities for physicians barely shifted. As many patients were moved to government-funded schemes, new "price sensitivities" were found in the demand for healthcare service, as customers would no longer be insulated from costs.(20)

The evidence from outside the US is less consolidated. Analysis of labour statistics and employment censuses from the UK market (21) show that healthcare employment declined suddenly in 2020 only to bounce back the year after, with dentists and nurses among the worst affected professions. Another British study looking at job ads during the pandemic (22) found more care work and nursing vacancies than in any other sector during lockdowns. Similar findings were reported for Serbia, where COVID-19 seems to have entrenched a continuous mismatch between supply and demand for physicians and nurses.(23) An online survey study from Iran (24) suggested that healthcare employment would have become less attractive because of COVID-19, as more health workers consider leaving. At the height of the first wave of the pandemic, there were reports (25) that Mexico, South Africa, and Zambia were recruiting doctors from abroad, as a surge in the demand for COVID-19 care was expected.

Brazil has been one of the world's most affected countries, with over 700,000 COVID-19 related deaths; its economy contracted by 3.9% in 2020, although it rebounded by 4.6% and 2.6% in the following years.(26) The country's unemployment rate is

For peer review only - http://bmjopen.bmj.com/site/about/guidelines.xhtml

currently at 9%, and the International Monetary Fund estimates that approximately 12 million jobs have been lost as a consequence of the pandemic, with a disproportionate impact among the lower income groups.(27)

The impact on labour markets in each country depended essentially by the epidemiological spread of the disease, by the lockdown measures implemented, and by the structure and stage of development of the economy.(4) Past economic recessions historically had little repercussions for physician employment, as in Brazil demand has always outstripped supply.(28)

Lockdown measures and restrictions were introduced patchily and by the previous government in Brazil,(29) with a view not to disrupt the formal and informal economy. Initial shortcomings in the availability of tests hampered the collection of epidemiological data for SARS-CoV-2 infections among health professionals, and for the first year of the pandemic there was no official testing protocol for the wider population or HWs.(30) Amid such an environment, elective medical procedures were suspended, but the vast majority of essential healthcare services kept functioning.(31) A recent study showed that public sector physicians' workload and earnings increased in Brazil during the first two years of the pandemic, while those of private doctors suffered.(32)

Brazil's healthcare system comprises a publicly funded Unified Health System (*Sistema Único de Saúde*, SUS) and a multiplicity of privately financed sub-sectors, including large, comprehensive private hospitals and smaller, outpatient care surgeries.(33) In the last 20 years, low-cost walk-in clinics in urban areas (*Clínicas Populares*, or People's Clinics) have started to provide out-of-pocket private services, mostly outpatient in nature.(34,35) Within SUS, provision of free-at-the-point-of-care services is often outsourced to private entities (social health organisations) that manage public hospitals and contract their own staff, including doctors. Private funds account for more than half (54%) of Brazil's health spending, including out-of-pocket medicines and private insurance premiums.(36) Access to such private services is funded through employment-related health insurance plans, with 24.2% of Brazil's population owning such private plans.(37)

Previous analysis of this very dataset showed that a substantial proportion of physicians in our sample were infected with SARS-CoV-2 virus during the first year of the pandemic, that the infection rate in Maranhão was almost twice that in São Paulo, as being a physician in Maranhão, younger, and having worked in a COVID-19 ward, were characteristics associated with the probability of infection.(38) We also found that workloads and earnings of public doctors increased the most in the first year of the epidemic, particularly in Maranhão. Although earnings remained broadly stable in the public sector, a third of public sector-only physicians in MA saw an increase in their earnings, while more than half of private ones saw a decrease in their earnings.(39) As for working modalities, in the same sample we also saw that telemedicine was employed by 1/3 of doctors as a modality to provide healthcare services, particularly for hospital-based in-patient services, more frequently in private clinics, in São Paulo more than in Maranhão.(40)

In this paper we use physicians' perceptions of changing employment opportunities in Brazil to gain an insight into health labour markets during COVID-19 in LMICs. The aim of this study is to contribute to the existing body of work on health labour markets in mixed health systems across the world; this would provide an evidence base for policies to mitigate the effects of future shocks on health workforces.

For peer review only - http://bmjopen.bmj.com/site/about/guidelines.xhtml

Methods

Methodological approach

As part of a wider study on the health workforce in two Brazilian states,(41) we conducted secondary analysis of a dataset from a representative cross-sectional survey on physicians' perceptions of the impact of COVID-19 on their health, earnings, and work routines.(32,42,43) Workers' perceptions have been used before in the economic literature to explore labour market dynamics in high-income contexts. (44)

In our survey, doctors were asked whether they had observed in the past two years: (a) an increase or a decline of job opportunities and vacancies in their workplace; (b) any change in the availability of doctors; and (c) any change in the remuneration of a 12-hour shift in their institution's Accidents and Emergency (A&E) ward (see the Survey Questionnaire in Supplementary Information 1). We interpreted the reported changes in (a) as proxies for changes in demand for doctors; responses on (b) as proxies for supply; and responses on (c) as a proxy for changes in the level of prices for healthcare services.

As our doctors worked in either public or private facilities, or both, we considered the doctors' perceptions of changes in their own sector of employment as particularly accurate. As a way of validating responses, we triangulated the perceptions of the entire sample with those from doctors working specifically in such sectors. We outline the limitations of such an approach in the Discussion.

Study settings and data collection

The original survey was carried out in one rich state (São Paulo) and one less developed one (Maranhão) in Brazil, with a view to capture the differential effects of economic recessions in diverse health markets.(37) São Paulo state is home to more than 46.6M people, has one of the country's highest per capita incomes, and 38% of its population is covered by private health schemes. The public health expenditure is also among the highest, estimated at US$ 360,28 per capita in 2018 (US$ 650 PPP), and its medical workforce includes 163,430 physicians (3.5 per 1,000 inhabitants, the third highest in the country). By contrast, Maranhão state is home to approximately 7.2M people, its per capita income is one-third of São Paulo's, and only 1% of the population is covered by private health schemes. In 2022, there were 8,743 physicians in Maranhão, that is, 1.22 per 1,000 – the second lowest rate among Brazilian states.(45)

Our survey was conducted between 16 February and 15 June 2021. The sample was drawn from the nominal listing of physicians registered with Brazil's Federal Council of Medicine in the two states. The study's overall sample was composed of 1,183 physicians, consisting of 632 from São Paulo and 551 from Maranhão. The sample was calculated based on the active physicians registered with the Federal Council of Medicine in the two states in 2021 (N=152,511 – 144,852 in São Paulo and 7,659 in Maranhão) and their key demographic characteristics. Proportional stratified sampling was used to replicate the physician distribution by gender, age, state, and residence. A larger proportion of Maranhão's universe of physicians was selected to allow for a sufficient N for the strata and doctor characteristics of interest. As the two states are very different—in São Paulo there are 30% of all the physicians in Brazil, and in Maranhão a little more than 1%—a proportional sample would have been too small to allow stratifications in Maranhão (see Table 1 in Online resources 2).

For peer review only - http://bmjopen.bmj.com/site/about/guidelines.xhtml

The survey was carried out by a specialised research institute (Datafolha Research Institute), under the technical supervision of the academic researchers. Primary data was collected via a telephone survey conducted in Portuguese by Datafolha data collectors, which included a field coordinator, experienced interviewers, and administrative staff responsible for checking missing data. Sample size calculations, sample selection, questionnaire design, substitution control, database assembly, and data analysis were performed by the authors of this paper.

Ethics approval statement

The original survey received approval from the Research Ethics Committees of the Federal University of Maranhão, Brazil (CEP UFMA 3.051.875) and from the Faculty of Medicine of the University of São Paulo, Brazil (CEP FMUSP 3.136.269). The doctors surveyed were informed about the objectives of the study, guaranteed the anonymity of the information provided, and their consent to participate obtained.

Patient and public involvement. Medical doctors as well as members of the public were consulted and participated in the design of the original version of the survey questionnaire. The questionnaire was then piloted by Datafolha in a subset of ten doctors in the two states, and a final version was elaborated following the feedback received.

Variables and data analysis

The interviews consisted of a 30-minute telephone questionnaire, containing 30 questions ranging from multiple, closed questions to interdependently concatenated and semi-open questions. The specific variables for this secondary analysis were constructed from the questions below from the Survey Questionnaire's Section III – Changes in the Labour Market (see Survey Questionnaire in Annex 1).

Table 1: Variables, survey questions, and categories used in the analysis

Variable	Survey question	Categories
Demand for physicians in the public sector (SUS)	Q.24 In your opinion, comparing to before the pandemic, at SUS____	1. There are fewer work opportunities<br>2. There have been no significant changes in work opportunities OR<br>3. There are more work opportunities?<br>9999. Does not know
Demand for physicians in private doctor surgeries	Q.26 In your opinion, comparing to before the start of the pandemic, in private doctor surgeries ____	1. There are fewer work opportunities<br>2. There have been no significant changes in work opportunities OR<br>3. There are more work opportunities?<br>9999. Does not know
Demand for physicians in large private hospitals	Q.28 In your opinion, comparing to before the start of the pandemic, in large private hospitals ____	1. There are fewer work opportunities<br>2. There have been no significant changes in work opportunities OR<br>3. There are more work opportunities?<br>9999. Does not know
Demand for physicians in private walk-in clinics	Q.30 In your opinion, comparing to before the start of the pandemic, in private walk-in clinics ____	1. There are fewer work opportunities<br>2. There have been no significant changes in work opportunities OR<br>3. There are more work opportunities?<br>9999. Does not know

For peer review only - http://bmjopen.bmj.com/site/about/guidelines.xhtml

Supply of physicians	Q.32 In your opinion, comparing to before the pandemic, the availability of new physicians to fill vacancies _______	1. Has decreased<br>2. There have been no significant changes in the number of medical professionals available OR<br>3. Has increased?<br>9999. Does not know
Prices of healthcare services	Q.34 In your opinion, comparing to before the pandemic, the amount paid to physicians for a 12h A&E shift _______	1. Has decreased.<br>2. There have been no significant changes in the amount paid to physicians in your specialty OR<br>3. Has increased?<br>9999. Does not know

Source: USP-UFMA-QMUL (2022).

For our analysis in this study, the prevalence and 95% CIs of variables related to physicians' perceptions on changes in job opportunities and availability of doctors were estimated for the two states in their facilities of practice (public hospitals-SUS, private doctor's surgeries, large private hospitals, and walk-in clinics), and stratified for public-only physicians, private-only ones, and dual practitioners (Table 1).

p-value were calculated based on chi-square tests. Statistically significant differences at the 5% confidence level were considered in the absence of overlapping 95% CIs. Prevalence and 95 CIs for such perceptions were also analysed and plotted by physicians' age groups (< 35 years, 35 to 50 years, ≥ 50 years), and by their specific involvement with COVID-19 services. The database developed in Excel by the Datafolha data collectors was exported to R-Studio version 4.1.3 for statistical treatment.(46)

Results

Our sample included 1,183 physicians, 551 from Maranhão and 632 from São Paulo, located in urban as well as rural areas (see the graphical map of physicians' locations in Fig. S1 in Online Resources 2).

Most physicians in our survey (58.3%) worked concomitantly in public and private healthcare services, with only 19.6% of them employed exclusively in the public (see Table S1 in Online Resources 2). Most of our physicians were deployed directly to the delivery of COVID-19 services, to COVID-19 wards or to COVID-19-specific outpatient care (63.4%). No significant difference was found in physicians' characteristics or employment across the two states.

Most doctors in our sample said job opportunities and vacancies in the public sector (SUS) increased during COVID-19 (54.9%, 95% CI 52.0-57.7). This was particularly evident among Maranhão doctors (65.0%, 95% CI 60.9-68.9). Opportunities and vacancies were also said to have increased in large private hospitals with in-patient care capacity (46.7%, 95% CI 43.9-49.6), although not as much as in SUS. Again, such positive perceptions were found to be more pronounced among Maranhão doctors (see Table 2).

Table 2: Physicians' perceptions on employment opportunities in public and private facilities, by sector of employment and state

For peer review only - http://bmjopen.bmj.com/site/about/guidelines.xhtml

Employment opportunities		Total (n=1,183)		São Paulo (n=632) 53.4% (50.6-56.3)		Maranhão (n=551) 46.6% (43.7-49.4)		p-value*
		%	CI95%	%	CI95%	%	CI95%	
Public system (SUS)	Increased	54.9	(52.0-57.7)	46.0	(42.2-49.0)	65.0	(60.9-68.9)	<0.001
	Reduced	15.6	(13.6-17.7)	18.4	(15.5-21.5)	12.3	(9.8-15.3)	
	No changes	22.5	(20.3-25.0)	24.4	(21.1-27.8)	20.5	(17.3-24.0)	
	Unknown	7.0	(5.7-8.6)	11.2	(9.0-13.9)	2.2	(1.2-3.7)	
Private doctor's practices	Increased	15.7	(13.7-17.9)	12.8	(10.4-15.6)	19.1	(15.9-22.5)	0.013
	Reduced	57.2	(54.4-60.0)	60.9	(57.1-64.7)	53.0	(48.8-57.1)	
	No changes	20.3	(18.1-22.6)	19.8	(16.8-23.0)	20.9	(17.6-24.4)	
	Unknown	6.8	(5.4-8.3)	6.5	(4.8-8.3)	7.0	(5.2-9.4)	
Large private hospitals	Increased	46.7	(43.9-49.6)	42.4	(38.6-46.3)	51.7	(47.6-55.9)	0.008
	Reduced	24.4	(22.0-26.9)	27.1	(23.7-30.6)	21.4	(18.1-25.0)	
	No changes	19.8	(17.6-22.1)	20.3	(17.3-23.5)	19.3	(16.1-22.7)	
	Unknown	9.1	(7.5-10.8)	10.2	(8.1-12.8)	7.6	(5.6-10.1)	
Walk-in clinics	Increased	25.2	(22.9-27.8)	24.7	(21.4-28.2)	26.0	(22.4-29.7)	<0.001
	Reduced	19.9	(17.7-22.2)	16.9	(14.2-20.0)	23.2	(19.9-26.6)	
	No changes	24.0	(21.6-26.5)	20.4	(17.4-23.7)	28.1	(24.5-32.0)	
	Unknown	30.9	(28.3-33.5)	38.0	(34.3-41.4)	22.7	(19.3-26.3)	

*Note: * p-value based on chi-square test. Source: USP-UFMA-QMUL (2022).*

For other private sector facilities, perceptions of changing employment opportunities were either negative or neutral, particularly for smaller private doctor's practices, where 57.2% of doctors declared opportunities to have actually decreased (95% CI 54.5-60.0). Such negative perceptions for smaller private clinics were especially acute in São Paulo (60.9%, 95% CI 57.1-64.7) (Table 2).

These perceptions were confirmed when separating the views of public and private sector doctors, as public doctors declared noticing increased opportunities in their own sector of employment by a larger margin (72.2%, 95% CI 66.1-77.7) and private sector doctors confirmed the reduction of opportunities in private doctor's practices by 62.7% (95% CI 56.7-68.4). For those doctors working concomitantly in public and private facilities – the dual practitioners – job opportunities increased in the public sector and in large private hospitals (56.4% and 46.3%, respectively), but reduced in smaller doctor's practices (62.5%, 95 CI 58.8-66.1) and stayed unchanged in walk-in clinics (see Table S2 in Supplementary Information 2).

Regarding the availability of new doctors to take up vacancies in specific sectors, there was a slight perception of increased availability among public health physicians (43.1%, 95 CI 36.4-49.1), although this was predominantly driven by the positive

For peer review only - http://bmjopen.bmj.com/site/about/guidelines.xhtml

perceptions of Maranhão doctors (51.4%, 95 CI 43.2-59.5) – among São Paulo doctors this perception was, in fact, neutral if not negative (see Table 3 below). Perceptions of increased availability of doctors were also recorded among dual practitioners in Maranhão (39.1%, 95 CI 37.8-48.2). For private health physicians, perceptions of positive changes were only significant for Maranhão doctors (43.5%, 95 CI 31.7-55.9).

Table 3: Perceptions on availability of doctors in each sector, by type of current public, private, and dual employment

Perception of availability of doctors	Total (n=1,183)		São Paulo (n=632)		Maranhão (n=551)		p-value
			53.4% (50.6-56.3)		46.6% (43.7-49.4)		
	%	CI95%	%	CI95%	%	CI95%	
**Public sector (SUS)**							
Increased	43.1	36.4-49.1	28.9	20.3-38.8	51.4	43.2-59.5	0.006
Reduced	32.3	25.3-37.2	37.8	28.3-48.1	26.8	20.0-34.5	
No changes	5.1	2.5-8.1	28.9	20.3-38.8	16.9	11.4-23.7	
Unknown	19.5	16.6-27.2	4.4	1.5-10.2	4.9	2.2-9.4	
**Private**							
Increased	38.7	31.8-43.5	35.7	29.3-42.5	43.5	31.7-55.9	0.475
Reduced	22.3	18.5-28.8	25.1	19.5-31.5	17.7	9.8-28.6	
No changes	7.9	5.8-12.7	29.6	23.6-36.3	32.3	21.6-44.5	
Unknown	31.1	24.9-36.0	9.5	6.0-14.2	6.5	2.2-14.6	
**Dual practice**							
Increased	39.1	36-43.3	36.2	31.2-41.3	43.0	37.8-48.2	0.057
Reduced	30.3	27.1-33.9	29.2	24.5-34.1	31.7	27-36.7	
No changes	5.9	4.6-8.2	26.8	22.3-31.7	20.7	16.7-25.2	
Unknown	24.7	20.7-27.0	7.8	5.4-11.1	4.6	2.8-7.2	
**Total**							
Increased	39.7	37-42.5	35.0	31.3-38.7	45.4	41.1-49.4	≥0.001
Reduced	29.0	26.5-31.6	29.1	25.7-32.7	28.7	25.2-32.7	
No changes	24.8	22.4-27.3	28.0	24.6-31.6	21.3	17.8-24.6	
Unknown	6.5	5.2-8.0	7.9	6.0-10.2	4.6	3.3-6.9	

** p-value based on chi-square test*
Source: USP-UFMA-QMUL (2022).

Such reported changes in availability of vacancies and doctors, however, were not reflected in the perception of changes in prices; the largest group of doctors across all sectors (39.1%, 95 CI 32.4-47.6) declared that remuneration for a 12-hour A&E shift stayed broadly unchanged during the pandemic (see Table S3 in Supplementary Information 2).

When disaggregating responses by age groups, younger doctors (aged 24–34) were the ones declaring increased job opportunities, particularly in the public sector (64%, 95 CI 58.1-68.1) (see Figure 1 below).

For peer review only

Figure 1: Proportion of doctors reporting increased job opportunities, by age group and type of facility

Source: USP-UFMA-QMUL (2022).

In private walk-in clinics, however, it was only the older doctors (>60 years) who reported significantly improved work opportunities (47.5%, 95 CI 39.9-55.1), with all the other age groups reporting either decreased or unchanged employment opportunities (Figure 1).

Doctors working directly with COVID-19 cases generally reported increased opportunities, particularly in public hospitals (65%, 95 CI 62.3-68.4) and in large private ones (55%, 95 CI 51.8-59.1) (see Figure 2). Walk-in clinics were the only exceptions, as in such facilities specialising in working with COVID-19 did not appear to significantly increase job opportunities (35.9%, 95 CI 32.1-40.0).

Figure 2: Proportion of doctors declaring increased opportunities, by specialisation in COVID-19 cases and type of facility

Source: USP-UFMA-QMUL (2022).

DISCUSSION

In our survey of labour market perceptions during COVID-19 among physicians in Brazil, we found that most doctors recounted increased job opportunities in the public sector, particularly in Maranhão state. For the private sector, perceptions were mixed, as increased opportunities were reported in large private hospitals but not in smaller practices or walk-in clinics. In regard to the availability of doctors, our survey recorded perceptions of small increases in Maranhão, particularly in the public sector. Remuneration of A&E shifts stayed broadly unchanged. Younger doctors were the ones declaring more job opportunities in public facilities. Older ones reported opportunities in walk-in clinics, particularly in Maranhão. Those doctors working directly with COVID-19 patients saw increases in SUS and large private hospitals, but not elsewhere.

We acknowledge that different doctors across the world experienced COVID-19 in a different way – from the frontline intensive care and infective diseases specialists who found themselves in the eye of the storm, to primary care specialists who transitioned to remote working and telemedicine, to surgeons who simply saw their non-essential procedures cancelled. However, our survey of physicians' perceptions in Brazil during COVID-19 suggests that job opportunities actually increased in the public sector and in large private hospitals. This is contrast to what was observed for US hospitals during the pandemic.(20)

Our interpretation is that, in countries like Brazil with publicly funded health systems, resources (and jobs) were proactively redirected toward COVID-19 cases. This would explain why SUS and large private hospitals with in-patient care capacity in Brazil appear to have experienced additional vacancies to meet the increased demand for COVID-19 care. Conversely, smaller private health facilities with mostly outpatient capacity may have temporally suspended some of their operations during the pandemic, in connection with the slow-down of demand for elective procedures. This

For peer review only - http://bmjopen.bmj.com/site/about/guidelines.xhtml

would be consistent with what was observed in public health systems in European countries.(22)

Labour markets in São Paulo and Maranhão appear to have been affected in different ways by the pandemic, with the latter state seeing an increase of services and employment opportunities, and São Paulo doctors witnessing reduced activities and opportunities. This may be due to the comparatively greater weight of public health services in Maranhão's health system. In São Paulo, the private healthcare sector is very developed,(47) with an estimated 86% of doctors engaging with it either exclusively or as dual practitioners.(48) We conclude that the São Paulo health labour market experienced during COVID-19 effects similar to the market in the US, while Maranhão's displayed features more like the European, Canadian, or Australian markets. Such effects were probably exacerbated by the scarcity of doctors in Maranhão,(49) who inevitably ended up taking more responsibilities (and risks) in the COVID-19 fight,(42)

Younger doctors reported increasing job opportunities across the board, particularly in the public sector and in Maranhão. We believe this reflects the decisions taken in Brazil – like in other countries – to deploy younger (and therefore less at risk) cadres to staff COVID-19 services and shelter more senior ones.(42) This would also be consistent with our findings on the increased opportunities in COVID-19 wards for younger doctors. We interpret the reported increase of opportunities for older doctors in walk-in clinics as an indication that more lucrative parts of the private market would still be primarily accessible to more senior, established physicians, with fewer opportunities for younger doctors.(15)

Reports of no changes in remuneration for A&E shifts appear to be at odds with standard economic theory that would predict an increase of prices in the presence of increased demand and stable supply of doctors.(50) On the one hand, we acknowledge that remuneration for 12-hour A&E shifts may not be a suitable bell weather for changes in equilibrium prices for medical services (see the Limitations section below). On the other hand, it may be possible that, in the short run, labour prices for medical services may prove inelastic,(51) particularly during a pandemic emergency.

Our findings have broader relevance for other countries and future epidemics. We showed that health labour markets do not necessarily shrink during outbreaks, and the impacts will depend on the balance of public and private services within national health systems. Public health systems (and physicians) around the world were a key pillar of policy response to the pandemic, opening new services, performing additional functions, and driving the clinical fight.(52) This inevitably poses questions on what the role of markets and the private sector should be during a public health emergency,(53) calling for a re-configuration of the complementarity of public and private functions, with a view to boosting pandemic preparedness in LMICs.(54)

Limitations

Our findings need to be interpreted in light of a number of limitations. First, we used physicians' perceptions of changes in vacancies and prices to gain an insight into the demand and supply of doctors during the pandemic in Brazil. Although workers' perceptions have been used before in the literature to explore labour market dynamics,(44) we acknowledge that an examination of employment data would be needed to validate our findings. We also asked our sampled physicians to report on changes that happened in the past two years, which could have been affected by recall

For peer review only - http://bmjopen.bmj.com/site/about/guidelines.xhtml

bias.(55) Furthermore, a substantial proportion of doctors in Brazil concomitantly work in different hospitals and services, and the public/private nature of the entity running the services is not always know to them;(45) as a result, some of the 'public physicians' may have been erroneously classified, as they may be in reality working for 'private entities' and vice versa. We acknowledge this may have to some extent distorted our findings on the reaction of private and public labour markets during the pandemic.

Secondly, our proxies for demand, supply, and price levels may have left too much room for interpretation, as some of our doctors struggled to distinguish between 'availability of job opportunities' and 'availability of doctors to fill vacancies'. Regarding this latter, we recognise that supply and recruitment difficulties would be better known by hiring administrators and executives responsible for running hospitals, rather than by physicians. Our question on changes in remuneration for A&E shifts was driven by the need to identify a price indicator for medical services that could be known to all the doctors surveyed.(56) However, we realise that not all doctors in Brazil carry out A&E shifts or necessarily have knowledge of changes in this price. Such an anomalous finding on price stability may be also be driven by erroneous measurement of supply, or by the complexity of adjusting prices for services in the public sector.

Finally, we recognise that Maranhão and São Paulo states present very particular configurations of labour market characteristics, organisation of health services, policies, and health workforces.(32,37,45) Therefore, our findings may not be entirely generalisable to other LMICs.

Conclusion

Limited evidence exists on health labour markets' impacts and adaptations during COVID-19, with some literature suggesting a reduction of services. We conducted a secondary analysis of survey data on physicians' perceptions around changing employment opportunities in one rich and one less developed state in Brazil in 2021, with the objective of gaining insights into health labour markets during epidemics in LMICs.

Most of our sampled doctors noticed increased job opportunities in the public sector, particularly in Maranhão state. For the private sector, perceptions were mixed, as increased opportunities were reported in large private hospitals but not in smaller clinics. Younger doctors perceived an increase of vacancies in public and in large private hospitals, while older ones reported opportunities in walk-in clinics, particularly in Maranhão. Those doctors working directly with COVID-19 patients saw increases in public and large private hospitals, but not elsewhere.

Our findings suggest that health labour markets may not necessarily shrink during epidemics, and that the impacts will depend on the balance of public and private services in national health systems. The complementary roles of health markets and of publicly and privately funded systems during a health emergency should be re-examined, with the objective of improving pandemic preparedness, particularly for LMICs.

For peer review only - http://bmjopen.bmj.com/site/about/guidelines.xhtml

Contributorship statement

GR contributed to conceptualisation, participated in data analysis, and drafted the manuscript. BdO contributed to conceptualisation, participated in data analysis, and reviewed the manuscript. AJC participated in data collection, participated in data analysis, and reviewed the manuscript. MS contributed to conceptualisation, and reviewed the manuscript. All the authors read and approved the final manuscript.

Competing interests

The authors declared no competing interests.

Funding

This study on whose findings this paper is based, received support from the Confap-MRC call for Health Systems Research Networks. Specifically, Bruno de Oliveira was funded by the Fundação de Pesquisa do Estado Maranhão (FAPEMA, Processo COOPI-00709/18) and Coordenação de Aperfeiçoamento de Pessoal de Nível Superior-Brasil (CAPES)—Finance Code 001, to the Graduate Program in Public Health. Giuliano Russo received an award from the NEWTON FUND, MEDICAL RESEARCH COUNCIL (UK), grant reference MR/R022747/1. Mário Scheffer and Alex Cassenote received an award from the Fundação de Amparo à Pesquisa do Estado de São Paulo (FAPESP-Brazil), 2017/50356-7. The funders had no role in the survey's design, implementation, or analysis.

Data sharing statement

All the data survey are available upon reasonable request.

For peer review only

REFERENCES

1. Wang H, Paulson KR, Pease SA, Watson S, Comfort H, Zheng P, et al. Estimating excess mortality due to the COVID-19 pandemic: a systematic analysis of COVID-19-related mortality, 2020–21. The Lancet. 2022 Apr 16;399(10334):1513–36.

2. Shang Y, Li H, Zhang R. Effects of Pandemic Outbreak on Economies: Evidence From Business History Context. Frontiers in Public Health [Internet]. 2021 [cited 2023 Mar 24];9. Available from: https://www.frontiersin.org/articles/10.3389/fpubh.2021.632043

3. Lee S, Schmidt-Klau D, Verick S. The Labour Market Impacts of the COVID-19: A Global Perspective. Ind J Labour Econ. 2020 Oct 1;63(1):11–5.

4. OECD. Labour market developments: The unfolding COVID-19 crisis [Internet]. Paris: OECD; 2021 Jul [cited 2023 Mar 24]. Available from: https://www.oecd-ilibrary.org/employment/oecd-employment-outlook-2021_7e1e1ad3-en

5. Russo G, Silva TJ, Gassasse Z, Filippon J, Rotulo A, Kondilis E. The impact of economic recessions on health workers: a systematic review and best-fit framework synthesis of the evidence from the last 50 years. Health Policy Plan. 2021 May 17;36(4):542–51.

6. Ellis RP, Martins B, Zhu W. Health care demand elasticities by type of service. J Health Econ. 2017 Sep;55:232–43.

7. Staiger DO, Auerbach DI, Buerhaus PI. Registered Nurse Labor Supply and the Recession — Are We in a Bubble? New England Journal of Medicine. 2012 Apr 19;366(16):1463–5.

8. Chen A, Sasso AL, Richards MR. Graduating into a downturn: Are physicians recession proof? Health Econ. 2018;27(1):223–35.

9. Lavergne MR, Hedden L, Law MR, McGrail K, Ahuja M, Barer M. The impact of the 2008/2009 financial crisis on specialist physician activity in Canada. Health Econ. 2018;27(11):1859–67.

10. Galbany-Estragués P, Nelson S. Migration of Spanish nurses 2009-2014. Underemployment and surplus production of Spanish nurses and mobility among Spanish registered nurses: A case study. Int J Nurs Stud. 2016 Nov;63:112–23.

11. Bambra C, Garthwaite K, Copeland A, Barr B. All in it together? Health inequalities, austerity, and the 'Great Recession' [Internet]. Health Inequalities. Oxford University Press; 2015 [cited 2020 Apr 29]. Available from: https://www.oxfordscholarship.com/view/10.1093/acprof:oso/9780198703358.001.0001/acprof-9780198703358-chapter-12

12. Russo G, Pires CA, Perelman J, Gonçalves L, Barros PP. Exploring public sector physicians' resilience, reactions and coping strategies in times of economic crisis; findings from a survey in Portugal's capital city area. BMC Health Serv Res. 2017 15;17(1):207.

For peer review only - http://bmjopen.bmj.com/site/about/guidelines.xhtml

13. McPake B, Maeda A, Araújo EC, Lemiere C, El Maghraby A, Cometto G. Why do health labour market forces matter? Bull World Health Organ. 2013 Nov 1;91(11):841–6.

14. Scheffler RM, Herbst CH, Lemiere C, Campbell J. Health Labor Market Analyses in Low- and Middle-Income Countries: An Evidence-Based Approach [Internet]. The World Bank; 2016 [cited 2023 Mar 24]. 286 p. (Directions in Development - Human Development). Available from: https://elibrary.worldbank.org/doi/abs/10.1596/978-1-4648-0931-6

15. McPake B, Scott A, Edoka I. Analyzing Markets for Health Workers: Insights from Labor and Health Economics [Internet]. The World Bank; 2014 [cited 2023 Mar 27]. 96 p. (Directions in Development - Human Development). Available from: https://elibrary.worldbank.org/doi/abs/10.1596/978-1-4648-0224-9

16. WHO. Health labour market analysis guidebook [Internet]. Geneva, Switzerland: Health Workforce Department, The World Health Organization; 2021 [cited 2023 Jan 10] p. 392. Available from: https://www.who.int/publications-detail-redirect/9789240035546

17. Causa O, Abendschein M, Luu N, Soldani E, Soriolo C. The post-COVID-19 rise in labour shortages [Internet]. Paris: OECD; 2022 Jul [cited 2023 Mar 21]. Available from: https://www.oecd-ilibrary.org/economics/the-post-covid-19-rise-in-labour-shortages_e60c2d1c-en

18. Bhandari N, Batra K, Upadhyay S, Cochran C. Impact of COVID-19 on Healthcare Labor Market in the United States: Lower Paid Workers Experienced Higher Vulnerability and Slower Recovery. Int J Environ Res Public Health. 2021 Apr 8;18(8):3894.

19. Matta S, Nicholas LH. Changes in Unemployment Among Health Care Workers Following the COVID-19 Pandemic. JAMA. 2022 Oct 25;328(16):1639–41.

20. Teasdale B, Schulman KA. Are U.S. Hospitals Still "Recession-proof"? N Engl J Med. 2020 Sep 24;383(13):e82.

21. Cantor J, Whaley C, Simon K, Nguyen T. US Health Care Workforce Changes During the First and Second Years of the COVID-19 Pandemic. JAMA Health Forum. 2022 Feb 25;3(2):e215217.

22. Arthur R. Studying the UK job market during the COVID-19 crisis with online job ads. PLOS ONE. 2021 May 27;16(5):e0251431.

23. Šantrić Milićević M, Mandić-Rajčević S, Stevanovic A. Health workers labor market before and during the Covid-19 pandemic: Health sector capacity of Serbia. European Journal of Public Health. 2022 Oct 1;32(Supplement_3):ckac131.283.

24. AL-Abrrow H, Al-Maatoq M, Alharbi RK, Alnoor A, Abdullah HO, Abbas S, et al. Understanding employees' responses to the COVID-19 pandemic: The attractiveness of healthcare jobs. Global Business and Organizational Excellence. 2021;40(2):19–33.

For peer review only - http://bmjopen.bmj.com/site/about/guidelines.xhtml

25. Shuchman M. Low- and middle-income countries face up to COVID-19. Nature Medicine. 2020 May 21;26(7):986–8.

26. The World Bank. Brazil Poverty and Equity Assessment : Looking Ahead of Two Crises [Internet]. Washington (DC): The World Bank; 2022 [cited 2023 Mar 23]. Report No.: 173787. Available from: https://documents.worldbank.org/en/publication/documents-reports/documentdetail/099230007062256153/P1746910e33a8407d0b0850b8f0f5bcf18c

27. Flamini V, Toscani F. The Short-Term Impact of COVID-19 on Labor Markets, Poverty and Inequality in Brazil. IMF Working Papers [Internet]. 2021 [cited 2023 Mar 23];(066). Available from: https://www.elibrary.imf.org/view/journals/001/2021/066/001.2021.issue-066-en.xml

28. Nogueira RP. [The socioeconomic crisis and the demand for health professionals in Brazil, 1976-1984]. Bol Oficina Sanit Panam. 1988 Jun;104(6):572–82.

29. Faria de Moura Villela E, López RVM, Sato APS, de Oliveira FM, Waldman EA, Van den Bergh R, et al. COVID-19 outbreak in Brazil: adherence to national preventive measures and impact on people's lives, an online survey. BMC Public Health. 2021 Jan 18;21(1):152.

30. Magno L, Rossi TA, Mendonça-Lima FW de, Santos CC dos, Campos GB, Marques LM, et al. Desafios e propostas para ampliação da testagem e diagnóstico para COVID-19 no Brasil. Ciênc saúde coletiva. 2020 Aug 28;25:3355–64.

31. Sott MK, Bender MS, da Silva Baum K. Covid-19 Outbreak in Brazil: Health, Social, Political, and Economic Implications. Int J Health Serv. 2022 Oct;52(4):442–54.

32. de Oliveira BLCA, Andrietta LS, Reis RS, de Carvalho RH de SBF, de Britto e Alves MTSS, Scheffer MC, et al. The Impact of the COVID-19 Pandemic on Physicians' Working Hours and Earnings in São Paulo and Maranhão States, Brazil. International Journal of Environmental Research and Public Health. 2022 Jan;19(16):10085.

33. OECD. OECD Reviews of Health Systems: Brazil 2021 [Internet]. OECD; 2021 [cited 2023 Mar 28]. (OECD Reviews of Health Systems). Available from: https://www.oecd-ilibrary.org/social-issues-migration-health/oecd-reviews-of-health-systems-brazil-2021_146d0dea-en

34. Godoy CV, Silva JB. O Fenômeno de expansão das "clínicas médicas populares" no bairro Centro de Fortaleza/Ceará/Brasil. In: XIV Colóquio Ibérico de Geografia [Internet]. Fotaleza, Ceará; 2014 [cited 2020 May 8]. Available from: http://www.lasics.uminho.pt/conferences/index.php/CEGOT/XIV_ColoquioIbericoGeografia/paper/view/1963

For peer review only

35. Lapa MR. As clínicas populares como uma alternativa à saúde no Brasil: um estudo de caso em uma clínica popular. 2014 [cited 2020 May 8]; Available from: http://www.repositorio.ufc.br/handle/riufc/33277

36. Gragnolati M, Lindelow M, Couttolenc B. Twenty Years of Health System Reform in Brazil. An Assessment of the Sistema Único de Saúde. Washington, DC: The World Bank; 2013. (Directions in development: Human Development). Report No.: http://dx.doi.org/10.1596/978-0-8213-9843-2.

37. Andrietta LS, Levi ML, Scheffer MC, Alves MTSS de B e, Oliveira BLCA de, Russo G. The differential impact of economic recessions on health systems in middle-income settings: a comparative case study of unequal states in Brazil. BMJ Global Health. 2020 Feb 1;5(2):e002122.

38. Russo G, Cassenote A, Oliveira BLCAD, Scheffer M. Demographic and professional risk factors of SARS-CoV-2 infections among physicians in low- and middle-income settings: Findings from a representative survey in two Brazilian states. PLOS Global Public Health. 2022 Oct 14;2(10):e0000656.

39. de Oliveira BLCA, Andrietta LS, Reis RS, de Carvalho RH de SBF, de Britto e Alves MTSS, Scheffer MC, et al. The Impact of the COVID-19 Pandemic on Physicians' Working Hours and Earnings in São Paulo and Maranhão States, Brazil. International Journal of Environmental Research and Public Health. 2022 Jan;19(16):10085.

40. Scheffer M, Cassenote A, de Britto e Alves MTSS, Russo G. The multiple uses of telemedicine during the pandemic: the evidence from a cross-sectional survey of medical doctors in Brazil. Globalization and Health. 2022 Sep 19;18(1):81.

41. MRC. UK Research and Innovation. 2018 [cited 2019 Jun 26]. How is the current crisis reshaping Brazil's health system? Strengthening health workforce and provision of services in São Paulo and Maranhão. Available from: https://gtr.ukri.org/projects?ref=MR%2FR022747%2F1

42. Russo G, Cassenote A, De Oliveira BLCA, Scheffer M. Demographic and professional risk factors of SARS-CoV-2 infections among physicians in low- and middle-income settings: Findings from a representative survey in two Brazilian states. PLOS Glob Public Health. 2022;2(10):e0000656.

43. Scheffer M, Cassenote A, de Britto E Alves MTSS, Russo G. The multiple uses of telemedicine during the pandemic: the evidence from a cross-sectional survey of medical doctors in Brazil. Global Health. 2022 Sep 19;18(1):81.

44. Dixon JC, Fullerton AS, Robertson DL. Cross-National Differences in Workers' Perceived Job, Labour Market, and Employment Insecurity in Europe: Empirical Tests and Theoretical Extensions†. European Sociological Review. 2013 Oct 1;29(5):1053–67.

45. Russo G, Levi ML, Seabra Soares de Britto E Alves MT, Carneiro Alves de Oliveira BL, de Souza Britto Ferreira de Carvalho RH, Andrietta LS, et al. How the 'plates' of a health system can shift, change and adjust during economic recessions: A

For peer review only

qualitative interview study of public and private health providers in Brazil's São Paulo and Maranhão states. PLoS One. 2020;15(10):e0241017.

46. R Core Team — European Environment Agency. R: A language and environment for statistical computing [Internet]. Boston, Mass: R Foundation for Statistical Computing; 2022 [cited 2022 Mar 9]. Available from: https://www.eea.europa.eu/data-and-maps/indicators/oxygen-consuming-substances-in-rivers/r-development-core-team-2006

47. Cruz JAW, da Cunha MAVC, de Moraes TP, Marques S, Tuon FF, Gomide AL, et al. Brazilian private health system: history, scenarios, and trends. BMC Health Services Research. 2022 Jan 10;22(1):49.

48. Miotto BA, Guilloux AGA, Cassenote AJF, Mainardi GM, Russo G, Scheffer MC. Physician's sociodemographic profile and distribution across public and private health care: an insight into physicians' dual practice in Brazil. BMC Health Serv Res. 2018 Apr 23;18(1):299.

49. Scheffer MC, Almeida C, Guilloux AGA, Guerra A, Miotto BA. Demografia médica no Brasil 2023 [Internet]. São Paulo (BRA): Faculdade de Medicina USP, Conselho Federal de Medicina; 2023 p. 125. Available from: https://amb.org.br/wp-content/uploads/2023/02/DemografiaMedica2023_8fev-1.pdf

50. Cockx B, Brasseur C. The demand for physician services. Evidence from a natural experiment. J Health Econ. 2003 Nov;22(6):881–913.

51. Baştav L. AN EMPIRICAL STUDY ON THE WAGE AND PRICE STICKINESS OF US ECONOMY (1990-2009) -. Ekonomik Yaklasim. 2011;22(81):45–64.

52. Haldane V, De Foo C, Abdalla SM, Jung AS, Tan M, Wu S, et al. Health systems resilience in managing the COVID-19 pandemic: lessons from 28 countries. Nat Med. 2021 Jun;27(6):964–80.

53. Williams OD. COVID-19 and Private Health: Market and Governance Failure. Development. 2020 Dec 1;63(2):181–90.

54. David Williams O, Yung KC, Grépin KA. The failure of private health services: COVID-19 induced crises in low- and middle-income country (LMIC) health systems. Global Public Health. 2021 Sep 2;16(8–9):1320–33.

55. Althubaiti A. Information bias in health research: definition, pitfalls, and adjustment methods. J Multidiscip Healthc. 2016 May 4;9:211–7.

56. Lee T, Propper C, Stoye G. Medical Labour Supply and the Production of Healthcare. Fiscal Studies. 2019;40(4):621–61.

For peer review only

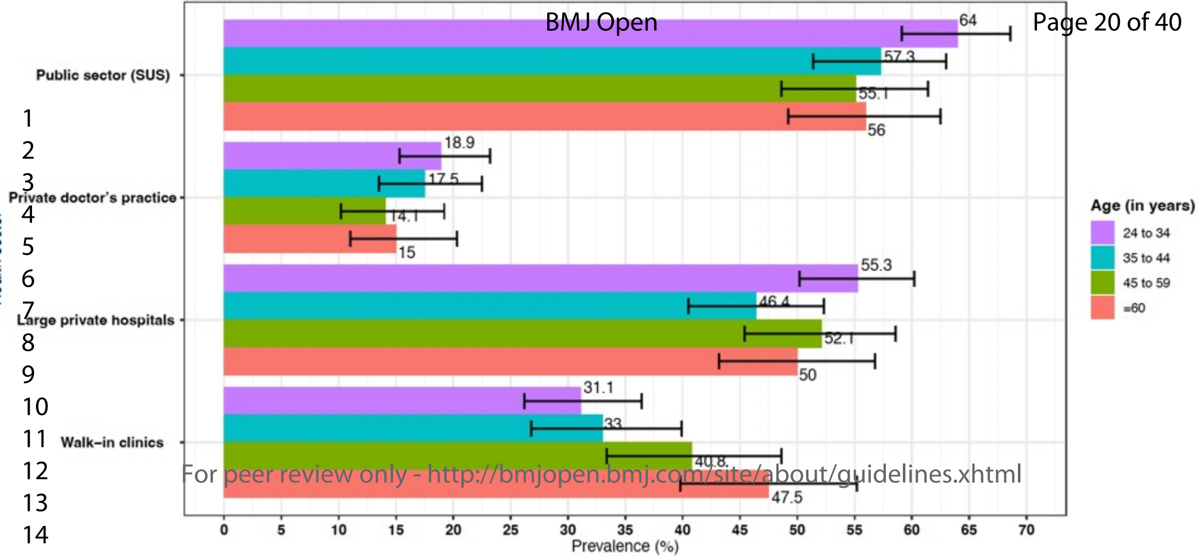

For peer review only - http://bmjopen.bmj.com/site/about/guidelines.xhtml

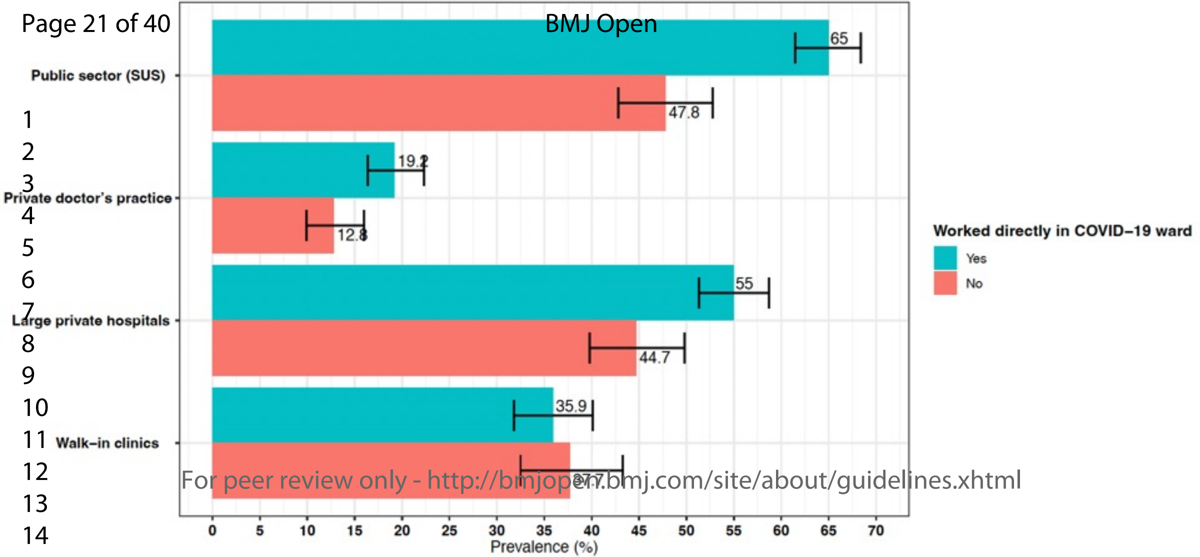

For peer review only - http://bmjopen.bmj.com/site/about/guidelines.xhtml

PM 745123 – SURVEY ON CORONAVIRUS IMPACT ON PHYSICIANS AND LABOUR MARKET IN SÃO PAULO AND MARANHÃO

					CPD NO.	
					CITY NO.	
INSPECTION	1. CHECKED	2. NO PHONE NUMBER	3. WRONG PHONE NUMBER	4. RESPONDENT NOT FOUND	SPOT NO.	
CLOSED-ENDED QUESTIONS SCORE				QTY:	SURVEY NO.	
OPEN-ENDED QUESTIONS SCORE				QTY:	SURVEY TAKER NO.	
TYPE	1. PROBABILISTIC	2. INTENTIONAL	3. ENLISTMENT		HOUR START	:
INSPECTOR NO.		CRITIC NO.		DATE: / /2020	HOUR END	:

Good morning/afternoon/evening. My name is ______. I work for Datafolha Research Institute and I am conducting a study, on behalf of ____ **(MENTION FROM LIST: IF SP > SAY USP; IF MA > SAY UFMA)** and the Federal Council of Medicine. Please, could I speak to Dr._______? **(PROCEED WITH THE NAME IN THE LIST. REPEAT FROM START IF THE CALL IS TRANSFERRED.)**

The survey we are conducting is about the medical work in Brazil within the context of the new coronavirus pandemic. The goal of this study is verifying the effects of the COVID-19 pandemic on the medical work in Brazil. This is an anonymous and confidential study that follows the standards of the Research Ethics Committee and the Medical Ethics Committee. Your name and answers will never be identified, and your data will remain stored only for the duration of the study. Do you agree to answer some questions?

1. Yes   2. No >> **END THE SURVEY**

For quality control purposes, this interview might be recorded.

GENDER   1. Male   2. Female

AGE What is your age? **(SPONTANEOUS ANSWER, ASK ONLY ONCE)**

[ ] **WRITE DOWN**

1. From 24 to 34 years
2. From 35 to 44 years
3. From 45 to 59 years
4. 60 years or older

QF.4 Do you currently work in the state of______ **(SÃO PAULO/ MARANHÃO – MENTION ACCORDING TO THE LIST)**

1. Yes
2. No **>> END THE SURVEY**

CRM:

[ ] **(GET FROM LIST)**

For peer review only - http://bmjopen.bmj.com/site/about/guidelines.xhtml

QF.3 In what year did you graduate? **(SPONTANEOUS ANSWER, ASK ONLY ONCE)**

(GET FROM LIST)

QF.1 Do you work as a physician or currently works with medicine or health?

1. Yes, full time (exclusivity contract) **PROCEED TO Q.36**
2. Yes, half time (partial dedication)
3. Does not work as physician / does not currently work with medicine or health

QF.2 (IF PF.1= 2 or 3) Did the decision of (giving partial dedication **IF QF1=2**) or (refrain from dedicating to medical work **IF PF1=3**) occur after March 2020, due to the pandemic?

1. Yes **PROCEED**
2. No **THANK THE RESPONDENT AND END THE SURVEY**

SET 1 – RESPONDENT CHARACTERIZATION

P.36 Have you been vaccinated, or do you intend to get vaccinated against COVID-19? **(SPONTANEOUS ANSWER, ASK ONLY ONCE)**

1. He/she is vaccinated
2. He/she has not been vaccinated, but intends to
3. He/she does not intend to get vaccinated

(SURVEY TAKER, READ THE FOLLOWING) THE COVID-19 PANDEMIC START IN BRAZIL IN MARCH 2020. I WILL ASK SOME QUESTIONS ABOUT YOUR WORK BEFORE AND AFTER THE START OF THE PANDEMIC.

P.1 Considering your current medical work, with its routine, format, volume of patients, and working hours, when compared to a usual level of work, before March 2020: **(READ PAUSEDLY UNTIL THE QUESTION MARK)**

1. It has not been impacted by the pandemic, performs the same work
2. Has been impacted, but resumed what used to be done before
3. Still suffers from impacts caused by the pandemic
4. The pandemic brought changes that will be permanently incorporated in his/her work
5. Physician work was interrupted due to the pandemic (is not currently working as a physician) **OR**
6. Retired from/definitively abandoned physician work due to the pandemic?

Q.2 Do you work, or did you work, on a regular basis, with medicine or health _______ ? **(READ EACH ITEM IN THE TABLE)** With any other area or type of activity that was not mentioned? **(IF YES, WRITE DOWN THE AREA OR ACTIVITY) (ENCOURAGED ANSWER AND SINGLE ANSWER PER LINE)**

For peer review only

Q.3 (FOR EACH Q.2=1) In this medical activity _____ **(READ THE ITEM)**, do you attend or did you attend, on a regular basis, only to patients with health insurance or private patients; attend/attended only SUS patients – (either at a public, philanthropic facility, Social Organization or another type of facility that provides services to SUS) OR attend /attended to both types of patients, work in both public and private sectors? **(ENCOURAGED ANSWER AND SINGLE ANSWER PER LINE)**

SHUFFLE ITEMS		Q.2 TYPE OF MEDICAL PRACTICE		Q.3 SECTOR OF WORK		
		YES	NO	PUBLIC	PRIVATE	BOTH
a.	With appointments at the office, ward or clinic?	1	2	1	2	3
b.	In the handling of diagnostic testing equipment?	1	2	1	2	3
c.	With surgeries in a hospital that require hospitalization and general/epidural/spinal anesthesia?	1	2	1	2	3
d.	With ward surgeries, without hospitalization and local anesthesia?	1	2	1	2	3
e.	Management, direction, service administration and institutional roles?	1	2	1	2	3
f.	Teaching or research roles?	1	2	1	2	3
**ALWAYS ASK AS LAST QUESTION**						
g.	Do you work in another sector or type of activity that was not mentioned? What___?	1	2	1	2	3

For peer review only

Q.4 (IF Q.3=2 or 3 IN AT LEAST ONE ITEM) You said that you work/worked in the private sector, attending to patients with health insurance or private patients. I will read a list of some workplaces in the private sector, and I would like to know in each one of them you work at or used to work at on a regular basis.

Do you work or did you work at _______ **(READ EACH ONE OF THE ITEMS)**? Is there any other place of the private sector that I have not mentioned? **(ENCOURAGED ANSWER AND SINGLE ANSWER PER ITEM)**

Q.5 (FOR EACH Q4=1) Approximately how many hours do you currently work at ____ **(READ EACH ITEM)** per week? **(ENCOURAGED ANSWER AND SINGLE ANSWER PER ITEM)**

SHUFFLE ITEMS	P.4 PRIVATE SECTOR WORK		P.5 HOURS/ WEEK
	YES	NO	(WRITE DOWN THE HOURS)

Datafolha
INSTITUTO DE PESQUISAS

				9996. Zero hours
a.	Own private office or shared with one or more colleagues?	1	2	______
b.	Private clinic or ward, where you provide care and services?	1	2	______
c.	Affordable private clinics<br><br>*(Example: Dr. Consulta, Cia da Consulta, Doutor Hoje for São Paulo; or Super Clínica, Mega Clínica, Super Clínica, Dr. Saúde for Maranhão)*	1	2	______
e.	Private hospital?	1	2	______
f.	Private diagnostic laboratory or private clinical analysis facility?	1	2	______
g.	Pharmaceutical industry?	1	2	______
h.	Medical sector of a company?	1	2	______
i.	Private university?	1	2	______
**ALWAYS ASK AS LAST QUESTION**				
k.	Is there any other place of the private sector that I have not mentioned? **(IF YES)** What? ___________ **(WRITE DOWN)**	1	2	______

Q.6 (IF Q.3=1 OR 3 IN AT LEAST ONE ITEM) You said that you work/worked in the public sector, attending to SUS patients or in public services or institutions. I will read a list of some workplaces in the public sector, and I would like to know in which one of them do you work at or used to work at on a regular basis.

Do you work or did you work at _______ **(READ EACH ONE OF THE ITEMS)**? Is there any other place of the private sector that I have not mentioned? **(ENCOURAGED ANSWER AND SINGLE ANSWER PER ITEM)**

Q.7 (FOR EACH Q.6=1) Approximately how many hours do you currently work at _____ **(READ EACH ITEM)** per week? **(ENCOURAGED ANSWER AND SINGLE ANSWER PER ITEM)**

**SHUFFLE ITEMS**	**Q.6 PUBLIC SECTOR WORK**		**P.7 HOURS/WEEK**
	**YES**	**NO**	**(WRITE DOWN HOURS)**<br><br>**9996. Zero hours**

For peer review only - http://bmjopen.bmj.com/site/about/guidelines.xhtml

**a.**	Primary health care, such as: *Basic Health Unit, Health Center, Family Health Program, Family Health Strategy*	**1**	**2**	______
**b.**	Ward services, such as: *Specialty Ward, AMA, mental health services – CAPs; HIV-Aids services; Hemocentre and Hemotherapy; Occupational Health Services*	**1**	**2**	______
**c.**	Urgent and emergency services, such as: *First aid services, emergency care, UPA (Emergency Unit), rescue, SAMU (Urgent Medical Assistance Service), pre-hospital care*	**1**	**2**	______
**d.**	Public hospital, such as: *SUS Hospital or an affiliated hospital that attends to SUS patients* *(Municipal, State or Federal Hospitals, University Hospitals, Charity Hospitals, Philanthropic Hospitals, Hospitals managed by Social Organization, Foundation or Hospital Authority)*	**1**	**2**	______
**g.**	Public university or public research institutions?	**1**	**2**	______
**h.**	Administrative or management services of public nature	**1**	**2**	______
**ALWAYS ASK AS LAST QUESTION**				
**k.**	Is there any other place of the public sector that I have not mentioned? **(IF YES)** What? __________ **(WRITE DOWN)**	**1**	**2**	______

ASK TO ALL RESPONDENTS

Q.8 I will read now some different regimes of employment or provision of medical services and I would like to know in which one of them you currently find yourself ______ **(ENCOURAGED ANSWER AND SINGLE ANSWER PER ITEM)**

Q.9 Before the pandemic, before March 2020, how was your employment regime ______ **(READ EACH ITEM)**?

**SHUFFLE ITEMS**		**Q.8 CURRENT EMPLOYMENT METHOD**		**Q.9 EMPLOYMENT METHOD BEFORE THE PANDEMIC**	
		**YES**	**NO**	**YES**	**NO**
**A.**	Statutory, civil servant?	1	2	1	2
**B**	CLT employment?	1	2	1	2
**C.**	Legal entity – PJ?	1	2	1	2
**D.**	Partner in a legal entity with other professionals?	1	2	1	2
**E.**	Cooperative?	1	2	1	2
**F.**	Freelancer with Self-employment income receipt?	1	2	1	2
**G.**	Contracts or individual agreements of another nature?	1	2	1	2

For peer review only - http://bmjopen.bmj.com/site/about/guidelines.xhtml

Datafolha
INSTITUTO DE PESQUISAS

Q.10 I will read some income ranges and I would like to know in what range were you in before the pandemic, before March 2020. Considering all your works and employments related to the practice of medicine that you had, approximately how much did you earn per month? **(ENCOURAGED ANSWER AND SINGLE ANSWER)**

1. Up to R$6,000
2. From R$6,001 to R$ 11,000
3. From R$ 11,001 to R$ 16,000
4. From R$ 16,001 to R$ 21,000
5. From R$ 21,001 to R$ 27,000
6. From R$ 27,001 to R$ 32,000
7. From R$ 32,001 to R$ 40,000
8. R$40,001 or more

9997. Refused to answer **(SPONTANEOUS ANSWER)**

SET II: CHANGES IN WORK OF RESPONDENTS CAUSED BY COVID-19 (both public and private sectors)

Q.11. Since March 2020, have you worked in activities/services directly related to COVID-19 by attending to infected/sick patients or in activities against the pandemic? **(IF YES)** Are you currently working in activities related to COVID-19? **(SPONTANEOUS AND SINGLE ANSWER)**

1. Have not worked and neither is working in any activity directly related to COVID-19.

   IF YES:
2. Is currently working in activities related to COVID-19.
3. Has worked in activities directly related to COVID-19 but is no longer working at the moment.

Q.12. (IF Q.11 = 2 or 3) In what kind of work, activity or place related to COVID-19 did you work/have you worked? **(ENCOURAGED ANSWER AND SINGLE ANSWER PER ITEM)**

**SHUFFLE ITEMS**		**Q.12**	
		**YES**	**NO**
**a.**	In hospital care of patients with COVID in ward or ICU	**1**	**2**
**b.**	In the care of suspicious or confirmed cases that do not require hospitalization	**1**	**2**
**c.**	In teleconsultation or other non-presential guidance or assistance activities related to COVID	**1**	**2**
**d.**	In scientific/academic research on COVID	**1**	**2**
**g.**	In health surveillance, committees or other governmental actions related to COVIC	**1**	**2**
**ALWAYS ASK AS LAST QUESTION**			
**h.**	Any other activity? What? ______ **(WRITE DOWN)**	**1**	**2**

For peer review only

Q.13 Considering a regular/habitual work week before the start of the pandemic, and your work after March 2020, has your number of worked hours per week increased, remained the same or decreased? **(ENCOURAGED ANSWER AND SINGLE ANSWER PER ITEM)**

1. Increased
2. Remained the same
3. Decreased

Q.14a (IF Q13=1) Approximately in how many weekly hours would you say that your work has increased since the start of the pandemic? **(SPONTANEOUS AND SINGLE ANSWER)**

________ hours **(WRITE DOWN)** **9999.** Does not know

Q.14b (IF Q13=3) Approximately in how many weekly hours would you say that your work has decreased since the start of the pandemic? **(SPONTANEOUS AND SINGLE ANSWER)**

________horas **(WRITE DOWN)** **9999.** Does not know

Q.15 Considering your monthly salary before March 2020 – at the start of the pandemic, has your salary been increased, remained the same or decreased? **(ENCOURAGED AND SINGLE ANSWER)**

1. Increased
2. Remained the same
3. Decreased

Q.16a (IF Q.15 = 1) How much, in Reais, has your monthly salary been increased? **(ENCOURAGED AND SINGLE ANSWER)**

(WRITE DOWN)

R$

9997. Refuses to answer
9999. Does not know

P.16b (SE P.15= 3) How much, in Reais, has your monthly salary been decreased? **(ENCOURAGED AND SINGLE ANSWER)**

(WRITE DOWN)

R$

9997. Refuses to answer
9999. Does not know

P.18 During the pandemic, how much do you think would be a fair amount for one hour of overtime work, in Reais? **(SPONTANEOUS AND SINGLE ANSWER)**

(WRITE DOWN)

R$

9997. Refuses to answer
9999. Does not know

For peer review only

Q.19 Were you diagnosed with COVID-19 at any moment? **(IF YES)** Were you asymptomatic, with mild symptoms or did you require hospitalization and/or ICU? **(ENCOURAGED AND SINGLE ANSWER)**

1. Yes, I was asymptomatic
2. Yes, I had mild symptoms
3. Yes, I required hospitalization and/or ICU.
4. I have not been diagnosed with COVID-19.

Q.20 (IF Q.19 ≠ 4) How many working days did you miss due to the COVID-19 diagnosis, up until now? **(SPONTANEOUS AND SINGLE ANSWER)**

(WRITE DOWN)

9996. None
9999. Does not know

Q.21. Do you_______ **(READ EACH ITEM)? (ENCOURAGED AND SINGLE ANSWER)**

**SHUFFLE ITEMS**		**Q.22**	
		**YES**	**NO**
**a.**	Carry out appointments and guidance to patients by telemedicine?	**1**	**1**
**b.**	Have work meetings by telemedicine?	**1**	**2**
**c.**	Have case discussions with colleagues by telemedicine?	**1**	**2**
**d.**	Perform prescriptions, certificates or reports by remote methods or telemedicine	**1**	**2**
**g.**	Do you prepare/annotate electronic medical records by telemedicine?	**1**	**2**
**h.**	Receive medical qualification or training by telemedicine	**1**	**2**
**ALWAYS ASK AS LAST QUESTION**			
**i.**	Perform any other activity by telemedicine? What? _____ **(WRITE DOWN)**	**1**	**2**

Q.22 (IF Q.21=1 IN AT LEAST OE ITEM) Considering your relationship with telemedicine/remote consultation, you: **(READ UNTIL THE QUESTION MARK) (ENCOURAGED AND SINGLE ANSWER)**

1. Had already been using this resource before the pandemic and kept using them,
2. Had never used this resource, but started using it due to the pandemic **OR**
3. Used the resource before, but no longer uses it since the beginning of the pandemic?
4. Had already used telemedicine occasionally to receive qualification, but has never used it with patients (SPONTANEOUS ANSWER)

Q.23 (IF Q.22 = 1 or 2) Currently, in your professional activities, how many hours do you dedicate yourself on a weekly basis to digital platforms/telemedicine/remote consultation? **(SPONTANEOUS AND SINGLE ANSWER)**

For peer review only - http://bmjopen.bmj.com/site/about/guidelines.xhtml

(ATTENTION, SURVEY TAKER, USE THE BOX WITH MINUTES ONLY IF THE RESPONDENT SPONTANEOUSLY SAYS THE TIME IN MINUTES AND IF HE/SHE MENTIONS LESS THAN 60 MINUTES)

**(WRITE DOWN in hours)**
**(WRITE DOWN in minutes)**<br><br>**(SPONTANEOUS ANSWER)**

9999. Does not know

For peer review only

SET III: CHANGES IN THE MEDICAL LABOR MARKET

Q.24 In your opinion, comparing to before the pandemic, at SUS_____ **(READ UNTIL THE QUESTION MARK – ENCOURAGED AND SINGLE ANSWER)**

1. There are fewer work opportunities
2. There have been no significant changes in work opportunities **OR**
3. There are more work opportunities**?**

9999. Does not know

Q.25a (IF Q.24=3) Using a score from one to ten, how much do you think that the work opportunities at SUS have been increased when compared to before the start of the pandemic?

ZERO										TEN	DOES NOT KNOW
9996	1	2	3	4	5	6	7	8	9	10	9999

Q.25b (IF Q.24=1) Using a score from one to ten, how much do you think that the work opportunities at SUS have been decreased when compared to before the start of the pandemic?

ZERO										TEN	DOES NOT KNOW
9996	1	2	3	4	5	6	7	8	9	10	9999

Q.26 In your opinion, comparing to before the start of the pandemic, at the private offices _____ **(READ UNTIL THE QUESTION MARK – ENCOURAGED AND SINGLE ANSWER)**

1. There are fewer work opportunities
2. There have been no significant changes in work opportunities **OR**
3. There are more work opportunities**?**

9999. Does not know

Q.27a (IF Q.26=3) Using a score from one to ten, how much do you think that the work opportunities at private offices have been increased when compared to before the start of the pandemic?

ZERO										TEM	DOES NOT KNOW
9996	1	2	3	4	5	6	7	8	9	10	9999

Q.27b (IF Q.27=1) Using a score from one to ten, how much do you think that the work opportunities at private offices have been decreased when compared to before the start of the pandemic?

ZERO										TEN	DOES NOT KNOW
9996	1	2	3	4	5	6	7	8	9	10	9999

Q.28 In your opinion, comparing to before the start of the pandemic, at the private hospitals _____ **(READ UNTIL THE QUESTION MARK – ENCOURAGED AND SINGLE ANSWER)**

1. There are fewer work opportunities
2. There have been no significant changes in work opportunities **OR**
3. There are more work opportunities**?**

9999. Does not know

Q.29a (IF Q.28=3) Using a score from one to ten, how much do you think that the work opportunities at private hospitals have been increased when compared to before the start of the pandemic?

ZERO										TEN	DOES NOT KNOW
9996	1	2	3	4	5	6	7	8	9	10	9999

Q.29b (IF Q.28=1) Using a score from one to ten, how much do you think that the work opportunities at private hospitals have been decreased when compared to before the start of the pandemic?

For peer review only - http://bmjopen.bmj.com/site/about/guidelines.xhtml

ZERO										TEN	DOES NOT KNOW
9996	1	2	3	4	5	6	7	8	9	10	9999

Q.30 In your opinion, comparing to before the start of the pandemic, at the affordable private clinics _____ **(READ UNTIL THE QUESTION MARK – ENCOURAGED AND SINGLE ANSWER)**

1. There are fewer work opportunities
2. There have been no significant changes in work opportunities **OR**
3. There are more work opportunities**?**

9999. Does not know

Q.31a (IF Q.30=3) Using a score from one to ten, how much do you think that the work opportunities at affordable private clinics have been increased when compared to before the start of the pandemic?

ZERO										TEN	DOES NOT KNOW
9996	1	2	3	4	5	6	7	8	9	10	9999

Q.31b (IF Q.30=1) Using a score from one to ten, how much do you think that the work opportunities at affordable private clinics have been decreased when compared to before the start of the pandemic?

ZERO										TEN	DOES NOT KNOW
9996	1	2	3	4	5	6	7	8	9	10	9999

Q.32 In your opinion, comparing to before the pandemic, the availability of physicians for hiring ________ **(READ UNTIL THE QUESTION MARK – ENCOURAGED AND SINGLE ANSWER)**

1. Has been decreased
2. There have been no significant changes in the number of medical professionals available **OR**
3. Has been increased**?**

9999. Does not know

Q.33a (IF Q.32=3) Using a score from one to ten, how much do you think that the availability of medical professionals for hiring has been increased, when compared to before the start of the pandemic?

ZERO										TEN	DOES NOT KNOW

Datafolha INSTITUTO DE PESQUISAS

9996	1	2	3	4	5	6	7	8	9	10	9999

Q.33b (IF Q.32=1) Using a score from one to ten, how much do you think that the availability of medical professionals for hiring has been decreased, when compared to before the start of the pandemic?

ZERO										TEN	DOES NOT KNOW
9996	1	2	3	4	5	6	7	8	9	10	9999

Q.34 In your opinion, comparing to before the pandemic, the amount paid to physicians for a 12h A&E shift in your specialty ________ **(READ UNTIL THE QUESTION MARK – ENCOURAGED AND SINGLE ANSWER)**

1. Has been decreased
2. There have been no significant changes in the amount paid to physicians in your specialty **OR**
3. Has been increased**?**

9999. Does not know

Q.35a (IF Q34=3) In terms of a percentage, how much do you think that the amount paid to physicians on duty in your specialty has been increased, when compared to before the pandemic? **(SINGLE ANSWER)**

(WRITE DOWN – 0% to 100%)

9999. Does not know

Q.35b (IF Q34=1) In terms of a percentage, how much do you think that the amount paid to physicians on duty in your specialty has been decreased, when compared to before the pandemic? **(SINGLE ANSWER)**

(WRITE DOWN– 0% to 100%)

9999. Does not know

I declare hereby that:
1. this survey was conducted in accordance with the instructions of the field supervision;
2. the information in this survey were correctly written down and they accurately correspond to the declarations of the respondent;
3. I am aware that the material collected by me is being or will be checked for quality control purposes;
4. I am aware that I must keep the confidentiality of the collected information;
5. I shall not reproduce this survey nor the information in it for my own use or third-party use.

NAME : ____

For peer review only

Datafolha INSTITUTO DE PESQUISAS

SIGNATURE: ____________________________ ID. Doc. No.: ___________________ Date: ____/ ___ / ___

For peer review only

Table S1: Physicians sample, but location and socio-economic characteristics

Physicians' sociodemographic characteristics	Proportion of total (n = 1,183)	São Paulo (n = 632) 53.4%	Maranhão (n = 551) 46.6%
Gender			
Male	56.2	54.1	58.6
Female	43.8	45.9	41.4
Age			
24 to 34	34.1	34.3	33.9
35 to 44	24.5	20.7	28.9
45 to 59	20.4	22.3	18.1
≥60	21.0	22.6	19.1
Geographical location of deployment			
Rural areas (Interior)	50.5	54.9	45.6
Urban areas around capital cities	49.5	45.1	54.5
Health sector of deployment			
Exclusively public	19.6	14.2	25.8
Exclusively private	22.1	31.5	11.3
Dual practice	58.3	54.3	63.0
Working directly with COVID-19 patients			
Yes	63.4	59.2	68.2
No	36.6	40.8	31.8

Table S2: Physicians' perceptions on employment opportunities in public and private facilities, by State

Employment opportunities		Total (n=1,181)		São Paulo (n=632) 53.4% (50.6-56.3)		Maranhão (n=551) 46.6% (43.7-49.4)	
		%	CI95%	%	CI95%	%	CI95%
**Public sector**							
SUS	Increased	72.2	(66.1-77.7)	67.4	(57.0-76.4)	75.2	(67.4-81.6)
	Reduced	11.9	(8.3-16.8)	18.6	(11.8-28.1)	7.8	(4.4-13.4)
	No changes	15.9	(11.7-21.2)	14.0	(8.2-22.8)	17.0	(11.7-24.1)
Private doctor's practice	Increased	18.8	(13.9-25.0)	13.0	(7.0-23.0)	22.2	(15.6-30.6)
	Reduced	55.4	(48.2-62.3)	65.2	(53.4-75.4)	49.6	(40.7-58.5)
	No changes	25.8	(20.1-32.5)	21.7	(13.6-32.8)	28.2	(20.8-37.0)
Large private hospitals	Increased	70.2	(63.5-76.1)	68.9	(57.7-78.3)	71.0	(62.4-78.2)
	Reduced	12.6	(8.7-18.0)	14.9	(8.5-24.7)	11.3	(6.9-18.1)

For peer review only

	No changes	17.2	(12.6-23.0)	16.2	(9.5-26.2)	17.7	(12.0-25.4)
Walk-in private clinics	Increased	40.9	(34.0-48.2)	45.9	(34.0-58.3)	38.3	(30.1-47.3)
	Reduced	21.0	(15.7-27.5)	21.3	(12.9-33.1)	20.8	(14.5-28.9)
	No changes	38.1	(31.4-45.4)	32.8	(22.3-45.3)	40.8	(32.5-49.8)
**Private sector**							
SUS	Increased	53.1	(46.4-59.6)	47.4	(39.7-55.3)	67.8	(55.1-78.3)
	Reduced	17.8	(13.3-23.5)	19.5	(14.0-26.4)	13.6	(7.0-24.5)
	No changes	29.1	(23.4-35.5)	33.1	(26.2-40.9)	18.6	(10.7-30.4)
Private doctor's practice	Increased	12.2	(8.7-16.7)	9.2	(5.9-14.0)	22.0	(13.4-34.1)
	Reduced	62.7	(56.7-68.4)	65.8	(58.9-72.1)	52.5	(40.0-64.7)
	No changes	25.1	(20.2-30.8)	25.0	(19.5-31.5)	25.4	(16.1-37.8)
Large private hospitals	Increased	49.6	(43.3-55.9)	45.2	(38.0-52.6)	62.7	(50.0-73.9)
	Reduced	24.2	(19.1-30.0)	26.0	(20.1-32.9)	18.6	(10.7-30.4)
	No changes	26.3	(21.1-32.2)	28.8	(22.6-35.9)	18.6	(10.7-30.4)
Walk-in private clinics	Increased	37.1	(30.3-44.5)	36.4	(28.6-45.0)	39.1	(26.4-53.5)
	Reduced	28.6	(22.4-35.7)	29.5	(22.3-37.8)	26.1	(15.6-40.3)
	No changes	34.3	(27.7-41.6)	34.1	(26.5-42.6)	34.8	(22.7-49.2)
**Dual practice**							
SUS	Increased	56.4	(52.6-60.1)	49.8	(44.4-55.3)	62.5	(57.3-67.5)
	Reduced	18.0	(15.3-21.1)	21.8	(17.6-26.6)	14.5	(11.1-18.6)
	No changes	25.6	(22.4-29.1)	28.3	(23.7-33.5)	23.0	(18.8-27.8)
Private doctor's practice	Increased	18.1	(15.4-21.2)	16.6	(12.9-21.0)	19.6	(15.7-24.2)
	Reduced	62.5	(58.8-66.1)	64.7	(59.4-69.7)	60.4	(55.1-24.5)
	No changes	19.3	(16.5-22.5)	18.7	(14.9-23.3)	19.9	(16.0-24.5)
Large private hospitals	Increased	46.3	(42.4-50.1)	43.4	(38.0-48.9)	49.1	(43.7-54.5)
	Reduced	32.2	(28.7-36.0)	36.1	(31.0-41.5)	28.5	(23.9-33.7)
	No changes	21.5	(18.5-24.8)	20.6	(16.5-25.4)	22.4	(18.2-27.2)
Walk-in private clinics	Increased	34.6	(30.4-39.1)	40.1	(33.6-47.0)	30.4	(25.1-36.2)
	Reduced	31.8	(27.7-36.2)	27.7	(22.0-34.3)	35.0	(29.5-41.0)
	No changes	33.5	(29.4-38.0)	32.2	(26.1-38.9)	34.6	(29.1-40.6)
**Total**							
SUS	Increased	59.0	(56.1-61.9)	51.9	(47.7-56.0)	66.4	(62.3-70.3)
	Reduced	16.7	(14.6-19.0)	20.7	(17.5-24.2)	12.6	(10.1-15.7)
	No changes	24.3	(21.8-26.9)	27.5	(23.9-31.3)	21.0	(17.7-24.6)
Private doctor's practice	Increased	15.8	(13.8-18.0)	13.7	(11.2-16.7)	20.5	(17.2-24.2)
	Reduced	57.4	(54.5-60.2)	65.1	(61.2-68.9)	57.0	(52.7-61.3)
	No changes	20.3	(18.1-22.7)	21.2	(18.0-24.6)	22.5	(19.1-26.3)

For peer review only

Hospitais Large private hospitals	Increased	51.4	(48.4-54.4)	47.3	(43.2-51.4)	56.0	(51.7-60.2)
	Reduced	26.9	(24.3-29.6)	30.2	(26.5-34.1)	23.2	(19.7-27.0)
	No changes	21.7	(19.4-24.3)	22.6	(19.3-26.2)	28.8	(17.5-24.6)
Walk-in private clinics	Increased	36.6	(33.3-39.9)	39.8	(35.1-44.7)	33.6	(29.2-38.2)
	Reduced	28.7	(25.7-31.9)	27.3	(23.1-31.9)	30.0	(25.9-34.6)
	No changes	34.7	(31.5-38.0)	32.9	(28.4-37.7)	36.4	(32.0-41.1)

Source: USP-UFMA-QMUL (2022)

Table S3: Perceptions on changes in remuneration for 12h A&E shift

Remuneration per shift		Total (n=1,181)		São Paulo (n=632) 53.4% (50.6-56.3)		Maranhão (n=551) 46.6% (43.7-49.4)	
		%	CI95%	%	CI95%	%	CI95%
Public	Increased	15.7	(11.5-21.2)	14.8	(8.7-24.1)	16.3	(11.0-23.4)
	Reduced	7.9	(5.0-12.2)	7.4	(3.4-15.2)	8.2	(4.6-14.0)
	No changes	39.3	(30.3-45.6)	44.7	(37.7-55.5)	43.2	(37.7-52.0)
	Unknown	37.1	(29.2-43.1)	33.1	(27.1-37.1)	42.3	(38.2-51.4)
Private	Increased	8.6	(5.5-13.2)	8.8	(5.3-14.2)	8.0	(3.2-18.8)
	Reduced	10.5	(7.0-15.4)	10.7	(6.8-16.5)	10.0	(4.4-21.4)
	No changes	40.9	(35.0-55.6)	40.5	(33.7-55.9)	42.0	(39.2-50.2)
	Unknown	40.0	(32.1-54.3)	37.3	(31.4-42.4)	42.3	(37.1-51.3)
Dual practice	Increased	12.4	(10.0-15.1)	12.8	(9.6-16.9)	11.9	(8.8-16.0)
	Reduced	14.9	(12.3-17.8)	16.2	(12.6-20.6)	13.5	(10.2-17.7)
	No changes	42.8	(39.2-46.1)	41.3	(35.8-45.7)	44.5	(39.5-49.0)
	Unknown	29.9	(21.3-33.4)	39.7	(33.6-43-4)	30.1	(25.5-36.1)
Total	Increased	12.3	(10.5-14.4)	11.9	(9.5-14.9)	12.7	(10.1-15.9)
	Reduced	12.6	(10.7-14.7)	13.4	(10.8-16.4)	11.7	(9.2-14.8)
	No changes	39.1	(32.4-47.6)	34.3	(28.9-48.1)	43.5	(36.6-48.1)
	Unknown	36.0	(28.7-42.6)	40.4	(32.4-45.8)	32.1	(25.2-37.8)

Source: USP-UFMA-QMUL (2022)

For peer review only

Figure S1: Geographical location of the surveyed physicians in São Paulo and Maranhão, Brazil

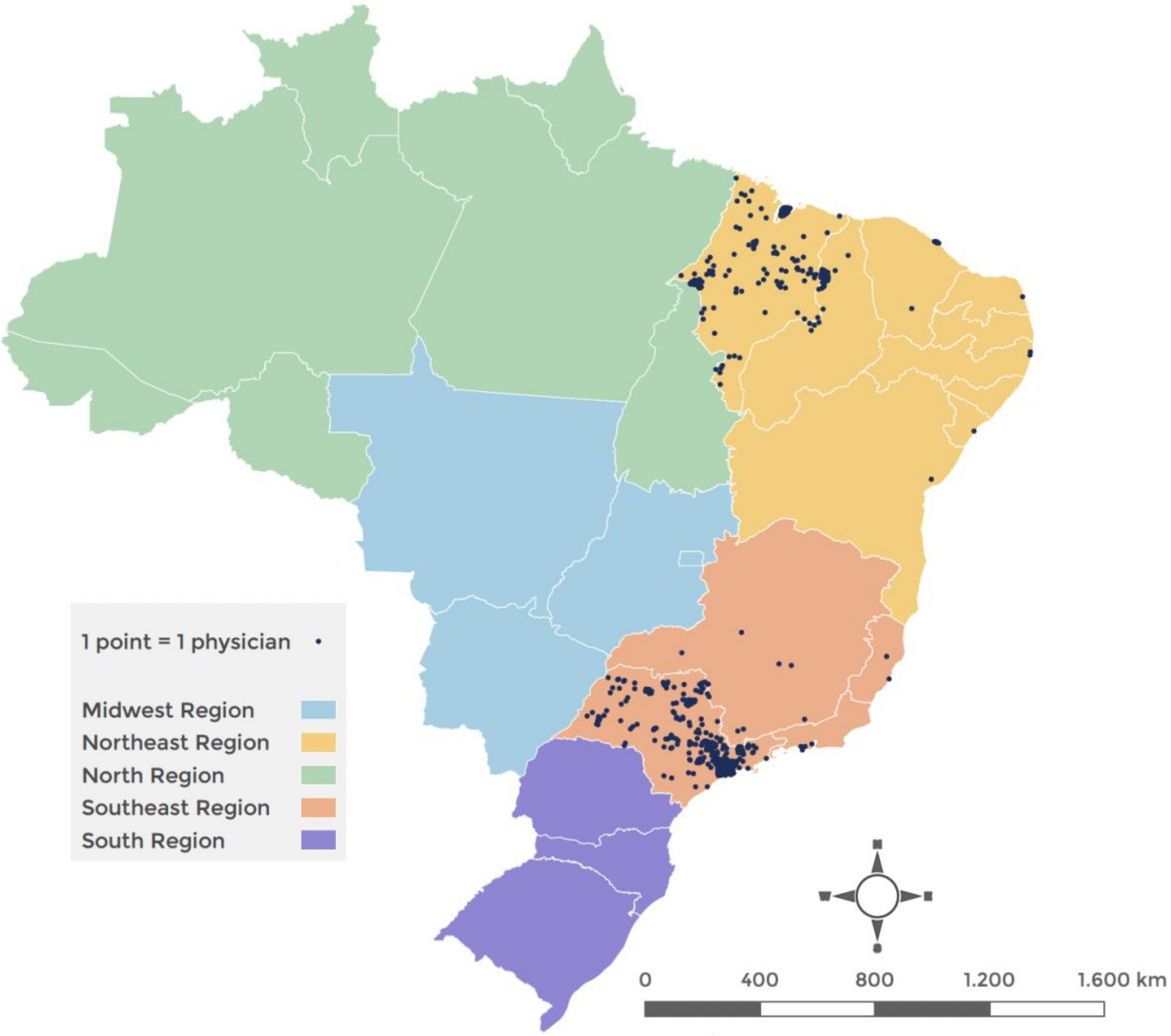

Source: Authors' own elaboration. The base layer map of Brazil is from the Brazilian Institute of Geography and Statistics.https://www.ibge.gov.br/geociencias/organizacao-do-territorio/malhas-territoriais/15774-malhas.html?=&t= downloads.

Checklist for Reporting Of Survey Studies (CROSS)

Section/topic	Item	Item description	Reported on page #
**Title and abstract**			
Title and abstract	1a	State the word "survey" along with a commonly used term in title or abstract to introduce the study's design.	Pag.1
	1b	Provide an informative summary in the abstract, covering background, objectives, methods, findings/results, interpretation/discussion, and conclusions.	Pag.1
**Introduction**			
Background	2	Provide a background about the rationale of study, what has been previously done, and why this survey is needed.	Pag. 3-4
Purpose/aim	3	Identify specific purposes, aims, goals, or objectives of the study.	Pag. 4
**Methods**			
Study design	4	Specify the study design in the methods section with a commonly used term (e.g., cross-sectional or longitudinal).	Pag. 4-5
Data collection methods	5a	Describe the questionnaire (e.g., number of sections, number of questions, number and names of instruments used).	Pag. 5-6
	5b	Describe all questionnaire instruments that were used in the survey to measure particular concepts. Report target population, reported validity and reliability information, scoring/classification procedure, and reference links (if any).	Pag.6
	5c	Provide information on pretesting of the questionnaire, if performed (in the article or in an online supplement). Report the method of pretesting, number of times questionnaire was pre-tested, number and demographics of participants used for pretesting, and the level of similarity of demographics between pre-testing participants and sample population.	Pag.5
	5d	Questionnaire if possible, should be fully provided (in the article, or as appendices or as an online supplement).	Online resources 1
Sample characteristics	6a	Describe the study population (i.e., background, locations, eligibility criteria for participant inclusion in survey, exclusion criteria).	Pag.5
	6b	Describe the sampling techniques used (e.g., single stage or multistage sampling, simple random sampling, stratified sampling, cluster sampling, convenience sampling). Specify the locations of sample participants whenever clustered sampling was applied.	Pag.5-6
	6c	Provide information on sample size, along with details of sample size calculation.	Pag.5
	6d	Describe how representative the sample is of the study population (or target population if possible), particularly for population-based surveys.	Pag.5

For peer review only

Survey administration	7a	Provide information on modes of questionnaire administration, including the type and number of contacts, the location where the survey was conducted (e.g., outpatient room or by use of online tools, such as SurveyMonkey).	Pag.5
	7b	Provide information of survey's time frame, such as periods of recruitment, exposure, and follow-up days.	Pag.5
	7c	Provide information on the entry process:<br>–>For non-web-based surveys, provide approaches to minimize human error in data entry.<br>–>For web-based surveys, provide approaches to prevent "multiple participation" of participants.	Pag.5
Study preparation	8	Describe any preparation process before conducting the survey (e.g., interviewers' training process, advertising the survey).	Pag.5
Ethical considerations	9a	Provide information on ethical approval for the survey if obtained, including informed consent, institutional review board [IRB] approval, Helsinki declaration, and good clinical practice [GCP] declaration (as appropriate)..5	Pag
	9b	Provide information about survey anonymity and confidentiality and describe what mechanisms were used to protect unauthorized access.	Pag.5
Statistical analysis	10a	Describe statistical methods and analytical approach. Report the statistical software that was used for data analysis.	Pag.6
	10b	Report any modification of variables used in the analysis, along with reference (if available).	N/A
	10c	Report details about how missing data was handled. Include rate of missing items, missing data mechanism (i.e., missing completely at random [MCAR], missing at random [MAR] or missing not at random [MNAR]) and methods used to deal with missing data (e.g., multiple imputation).	N/A
	10d	State how non-response error was addressed.	N/A
	10e	For longitudinal surveys, state how loss to follow-up was addressed.	N/A
	10f	Indicate whether any methods such as weighting of items or propensity scores have been used to adjust for non-representativeness of the sample.	N/A
	10g	Describe any sensitivity analysis conducted.	N/A
**Results**			
Respondent characteristics	11a	Report numbers of individuals at each stage of the study. Consider using a flow diagram, if possible.	Pag.7
	11b	Provide reasons for non-participation at each stage, if possible.	N/A

For peer review only

	11c	Report response rate, present the definition of response rate or the formula used to calculate response rate.	Pag.7-8
	11d	Provide information to define how unique visitors are determined. Report number of unique visitors along with relevant proportions (e.g., view proportion, participation proportion, completion proportion).	Pag.7
Descriptive results	12	Provide characteristics of study participants, as well as information on potential confounders and assessed outcomes.	Pag.7
Main findings	13a	Give unadjusted estimates and, if applicable, confounder-adjusted estimates along with 95% confidence intervals and p-values.	Pag.7-9
	13b	For multivariable analysis, provide information on the model building process, model fit statistics, and model assumptions (as appropriate).	N/A
	13c	Provide details about any sensitivity analysis performed. If there are considerable amount of missing data, report sensitivity analyses comparing the results of complete cases with that of the imputed dataset (if possible).	N/A
**Discussion**			
Limitations	14	Discuss the limitations of the study, considering sources of potential biases and imprecisions, such as non-representativeness of sample, study design, important uncontrolled confounders.	Pag.11
Interpretations	15	Give a cautious overall interpretation of results, based on potential biases and imprecisions and suggest areas for future research.	Pag.9-10
Generalizability	16	Discuss the external validity of the results.	Pag.11
**Other sections**			
Role of funding source	17	State whether any funding organization has had any roles in the survey's design, implementation, and analysis.	Pag.2
Conflict of interest	18	Declare any potential conflict of interest.	Pag.2
Acknowledgements	19	Provide names of organizations/persons that are acknowledged along with their contribution to the research.	Pag.2

For peer review only
